# Multiple rare and common variants in *APOB* gene locus associated with oxidatively modified low-density lipoprotein levels

**DOI:** 10.1371/journal.pone.0217620

**Published:** 2019-05-31

**Authors:** Eleonora Khlebus, Vladimir Kutsenko, Alexey Meshkov, Alexandra Ershova, Anna Kiseleva, Anton Shevtsov, Natalia Shcherbakova, Anastasiia Zharikova, Vadim Lankin, Alla Tikhaze, Irina Chazova, Elena Yarovaya, Oksana Drapkina, Sergey Boytsov

**Affiliations:** 1 Federal State Institution National Medical Research Center for Preventive Medicine of the Ministry of Healthcare of the Russian Federation, Moscow, Russia; 2 Moscow Institute of Physics and Technology (State University), Moscow, Russia; 3 Lomonosov Moscow State University, Moscow, Russia; 4 Federal State Budget Organization National Medical Research Center of Cardiology of the Ministry of Healthcare of the Russian Federation, Moscow, Russia; University of Texas Health Science Center at San Antonio, UNITED STATES

## Abstract

Oxidatively modified low-density lipoproteins (oxLDL) play an important role in the occurrence and progression of atherosclerosis. To identify the genetic factors influencing the oxLDL levels, we have genotyped 776 DNA samples of Russian individuals for 196,725 single-nucleotide polymorphisms (SNPs) using the Cardio-MetaboChip (Illumina, USA) and conducted genome-wide association study (GWAS). Fourteen common variants in the locus including *APOB* gene were significantly associated with the oxLDL levels (*P* < 2.18 × 10^−7^). These variants explained only 6% of the variation in the oxLDL levels. Then, we assessed the contribution of rare coding variants of *APOB* gene to the oxLDL levels. Individuals with the extreme oxLDL levels (48 with the lowest and 48 with the highest values) were selected for targeted sequencing of the region including *APOB* gene. To evaluate the contribution of the SNPs to the oxLDL levels we used various statistical methods for the association analysis of rare variants: WST, SKAT, and SKAT-O. We revealed that both synonymous and nonsynonymous SNPs affected the oxLDL levels. For the joint analysis of the rare and common variants, we conducted the SKAT-C testing and found a group of 15 SNPs significantly associated with the oxLDL levels (*P* = 2.14 × 10^−9^). Our results indicate that the oxLDL levels depend on both common and rare variants of the *APOB* gene.

## Introduction

Atherosclerosis is a complex multifactorial disease that is a major cause of cardiovascular disorders and a leading cause of mortality in developed countries. Over the last few decades, it was also suggested that the low density lipoprotein (LDL) oxidation plays an important role in the development and progression of atherosclerosis and its complications [1–4].

Under oxidative stress, accompanying the atherosclerosis development, the oxidized (lipoperoxides-containing) and/or oxidatively modified (containing the apoprotein modified by the secondary oxidation products—dicarbonyls) LDL particles accumulate in plasma [3, 4]. The oxidatively modified LDL (unlike oxidized LDL) is actively captured by macrophages, leading to the formation of the lipid enriched foam cells [5].

Previously, by performing the genome-wide association study (GWAS) in the Finnish population, it was shown that the oxLDL levels can be genetically determined [6]. However, human GWASs have important limitations. GWASs are typically focused on the common genetic variants with the minor allele frequency (MAF) greater than 0.05 and even the very large GWASs explain only a small fraction of the estimated heritability [7]. In the previously mentioned GWAS, the top significant SNP (rs676210) explained only 11% of the variation in the oxLDL [6]. In the current research, we have replicated the significant associations in an independent study of a Russian cohort and explained only 6% of the variation in the oxLDL levels.

One of the possible explanation of the ‘missing heritability’ is given by an underestimation of rare variants [8–10]. An association study of the low-frequency and rare coding variants with the blood lipids and coronary heart disease was performed previously [11]. The contribution of the rare variants to the low levels of high density lipoprotein (HDL) cholesterol was studied [12]. Targeted sequencing studies in subjects with the low cholesterol levels detected the rare mutations in *LDLR* [13], *PCSK9* [14], and *NPC1L1* [15] genes. Also, an effect of common and rare gene variants on plasma LDL cholesterol was assessed [16]. However, the distinct role of rare variants in the LDL oxidation has yet to be understood as well as their potential role in atherosclerosis. Therefore, to fill the heritability gap, we have verified the hypothesis that the rare (MAF<0.01) or low-frequency variants (MAF 0.01–0.05), which are not well covered by GWASs and not easily imputed, are also associated with the oxLDL levels.

To assess the contribution of the SNPs with the low MAF, the variants aggregation tests were developed. Instead of testing each SNP individually, these tests evaluate the cumulative effects of variants [17]. In our work, to find an association between the genetic variants and the oxLDL levels, we used the weighted-sum test (WST) [18], approaches based on the sequence kernel association test (SKAT) [19–22] and the adaptive combination of *P*-values method (ADA) [23, 24].

Here, we present the first results on a GWAS with oxLDL levels in Russian cohort. Furthermore, we report the association between the oxLDL levels and coding variants identified in *APOB* gene in the groups of patients with the extreme low and high oxLDL levels. Thus, for the first time in literature, we show the contribution of the common SNPs, as well as of the low-frequency and rare variants, to the oxLDL levels.

## Materials and methods

For more information, see S1 Appendix.

### Study subjects

**Fig 1:**
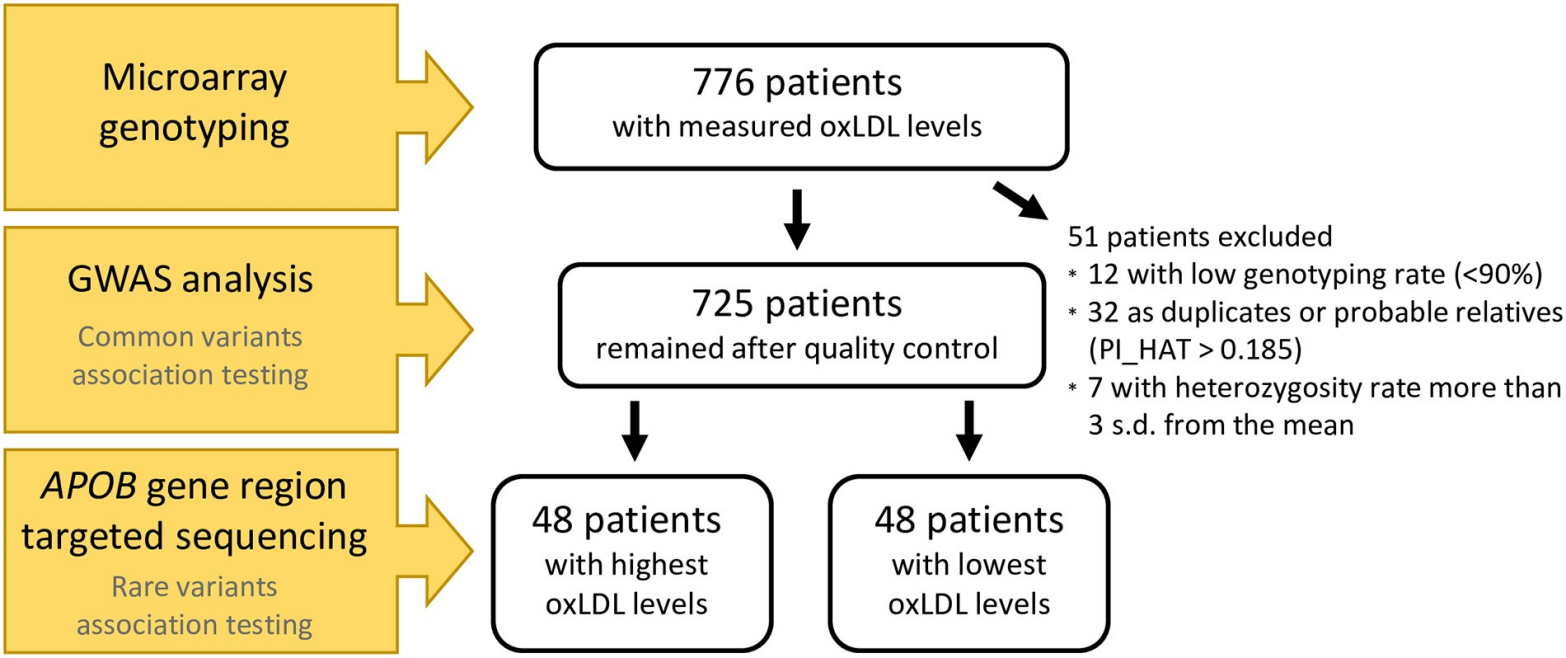
Cohort diagram indicating a number of patients participating into different steps of research.

Study subjects were recruited from the Russian study “Approbation and implementation of new approaches to prevention, diagnosis, and treatment of atherosclerosis in outpatient settings by the example of the Western Administrative District of Moscow” from August to December 2009. OxLDL levels were measured for 776 patients with various cardiovascular risk according to the SCORE [25], these patients were selected for genotyping and GWAS analysis. DNA samples from 48 individuals with the highest oxLDL levels and 48 individuals with the lowest oxLDL levels out of total cohort were selected for targeted sequencing (Fig 1). The study was approved by the Russian Cardiology Research and Production Complex, A.L. Myasnikov Institute of Clinical Cardiology (Committee on the Ethics issues in clinical cardiology, protocol No.144, 27 April 2009). Written informed consent was obtained from all participants after approval by the ethical committee.

### Laboratory tests

Blood samples were taken from each of the subjects in the morning after he or she had fasted overnight. Circulating serum oxLDL levels were assayed by Oxidized LDL ELISA kit (Mercodia, Sweden) according to the protocol [26].

Atherosclerosis progression is accompanied by oxidative stress, while malondialdehyde (MDA) and other low molecular weight dicarbonyls can accumulate in blood plasma, including MDA homologue glyoxal and MDA isomer methylglyoxal [4]. Glyoxal and methylglyoxal, like MDA, can cause the atherogenic modification of LDL [4, 26, 27]. Previously we have determined that by using Oxidized LDL ELISA kit (Mercodia, Sweden) we can measure not any oxidized LDL, but mainly MDA-modified LDL (S1 Fig) [28]. Further under oxidatively modified LDL (oxLDL) we mean only these MDA-modified LDL.

Total cholesterol (TC), triglycerides (TG), HDL, apolipoprotein A1 (ApoA1), apolipoprotein B (ApoB), high-sensitivity C-reactive protein (CRP), and lipoprotein (a) levels were measured using an automatic biochemistry analyzer ARCHITECT c8000 (Abbott Laboratories, USA). LDL was calculated according to the Friedewald formula [29] and in the case of TG>4.5 mmol/l LDL was estimated by the direct method using the same analyzer ARCHITECT c8000.

### Measurement of intima-medial thickness and plaque parameters

High-resolution B-mode ultrasonography was performed with a 11-3 MHz linear-type probe (PHILIPS iE33 ultrasound system, Philips Inc., Eindhoven, Netherlands). All measurements were taken in the common carotid artery, carotid bulb and proximal segment of internal carotid artery. Three different longitudinal views (anterior oblique, lateral, and posterior oblique) of both carotid systems and transverse views of all plaques were obtained. A more detailed procedure was described in an earlier publication [30]. The individual value of mean intima-medial thickness (mean-IMT) was the mean of mean-IMTs of the right and left carotid arteries.

The presence of atherosclerotic plaque was estimated at 6 sites of carotid pool: the whole length of both common carotid arteries, both bifurcations, and both internal carotid arteries. Plaque was defined as a focal structure that encroached into the arterial lumen by at least 0.5 mm or 50% of the surrounding IMT value or demonstrated a thickness of ≥ 1.5 mm as measured from the media-adventitia interface to the intima-lumen interface [31]. Plaque number was defined as the sum total of the plaques. An individual value of total stenosis was defined as a sum of maximum reductions in the percent diameter stenosis of all carotid plaques.

### Microarray-based genotyping and quality control

Genotyping was performed by using Cardio-MetaboChip (Illumina, USA), designed to test 196,725 SNPs. Quality control for patients and SNPs was conducted by PLINK (v 1.07) [32]. SNPs which passed quality control had parameters: call rate >0.95, MAF >0.05, Hardy-Weinberg equilibrium *P* > 1.0 × 10^−5^. Also, we excluded duplicates or probable relatives based on pairwise identity by state (PI_HAT > 0.185) and samples with heterozygosity rates more than 3 s.d. from the mean. To determine whether our sequence variations were caused by the population stratification, we assayed genotyping data using the principal component analysis (PCA). The PCA revealed no evidence of differences in genetic ancestry between samples (S2 Fig).

### Target enrichment and DNA sequencing

Targeted sequencing was performed using the TargetSeq Custom Enrichment Kit (Thermo Fisher Scientific, USA) on the SOLiD 5500W system (Thermo Fisher Scientific, USA) according to the manufacturer’s protocol. TargetSeq Custom Enrichment Kit was designed to target the region containing the complete genomic sequence of the *APOB* gene in locus 2p24-p23 (chr2: 20996301-21494945; GRCh37/hg19 reference human genome). The kit consists of 536 fragments accounting for a total of 391 833 bp. Unique probes were designed using the Sequence Search and Alignment by Hashing Algorithm (SSAHA) [33]. Capture design coordinates are provided in S1 Dataset.

### Variant calling, postprocessing, and multiple alignment

Mapping and variant calling were performed with LifeScope Genomic Analysis Software for SOLiD Next-Generation Sequencing (GRCh37/hg19 reference human genome). We used Samtools [34] for duplicate reads removal. To get coverage data we used BedTools [35]. We filtered variants using 10x coverage threshold. Each base with low-quality (Phred score < 30) sequence was also removed. Value 30 means that read assigned a Phred mapping quality with this score has a 1 in 1000 chance of being misaligned [36]. We used ANNOVAR software [37] for SNPs annotation. Functional effects of the detected variants were assessed with the SIFT [38] and PolyPhen-2 [39] algorithms. Multiple protein alignment was obtained using MUSCLE [40] and visualized using Jalview [41].

### Statistical analysis

Univariable statistical analysis was performed with Statistica v.8.0. *P*-value of less than 0.05 was considered to be statistically significant. Data were presented as median (25th–75th percentile). *P*-value for quantitative parameters was calculated using a non-parametric Mann-Whitney test. *P*-value for quality parameters was calculated using Yates’ corrected *χ*^2^ test. If the sample size included five subjects or fewer, a two-tailed Fisher’s exact test was used. Correlation analysis was performed by Spearman’s rank correlation test. For estimation of variation in the oxLDL levels non-adjusted R^2^ was used.

Association of common SNPs, obtained by microarray genotyping, with oxLDL levels in GWAS was tested using the PLINK software (v 1.07) [32]. Also, we examined the SNPs association with the other parameters: oxLDL/LDL and oxLDL/ApoB ratios, TC, TG, LDL, HDL, CRP, ApoA1 and ApoB.

For the analysis of rare and low-frequency variants obtained by targeted sequencing, we used weighted-sum test (WST) [18] and sequence kernel association test (SKAT) [19]. Also, we used optimal SKAT (SKAT-O) method which is based on combination of burden test and SKAT [20]. As covariates we used age, sex, smoking status, body mass index, waist, HDL, CRP, lipoprotein (a), hypertension, myocardial infarction, diabetes mellitus, stroke, and statins use. For the joint analysis of rare, low-frequency and common variants, we applied combined SKAT (SKAT-C) [21]. For the analysis of rare and low-frequency variants by WST we used the custom script in programming language R, for methods SKAT, SKAT-O and SKAT-C R-package [42]. Variants selection was performed by using results of adaptive combination of *P*-values (ADA) method [24] and SKAT backward elimination (BE-SKAT) test [22]. To select a subgroup of associated variants, as suggested at [43], we used the elastic net [44] from R-package [45] with AUC-ROC-based cross-validation.

## Results and discussion

### GWAS for finding variants affecting the oxLDL levels

We studied 776 DNA samples of Russian individuals with measured oxLDL levels for 196,725 SNPs genotyped by the Cardio-MetaboChip (Illumina, USA). Based on the quality control, we selected 725 patients and 101,704 SNPs. Clinical and laboratory characteristics of the patients are shown in Table 1. Under oxLDL we mean the MDA-modified LDL [28].

**Table 1:** Characteristics of 725 patients cohort and individuals with low and high oxLDL levels.

Parameter	Total cohort, n = 725	Low oxLDL levels group, n = 48	High oxLDL levels group, n = 48	*P*-value*
Age, years	57 (51-63)	59 (50-64)	56 (51-62)	0.703
Men, n (%)	206 (28.4)	21 (43.8)	17 (35.4)	0.531
Smoking, n (%)	108 (14.9)	7 (14.6)	12 (25.0)	0.306
BMI, kg/m2	28.7 (25.8-32.2)	28.7 (24.9-31.2)	28.3 (26.1-32.0)	0.613
Waist, cm	93 (84-102)	94 (84-101)	90 (85-102)	0.946
Total cholesterol, mmol/l	5.90 (5.17-6.88)	4.25 (3.90-4.88)	7.66 (7.01-8.25)	1.17 × 10^−14^
Triglycerides, mmol/l	1.60 (1.14-2.19)	1.22 (0.84-1.71)	2.20 (1.67-2.91)	1.55 × 10^−7^
LDL, mmol/l	3.60 (2.92-4.39)	2.31 (1.90-2.60)	4.86 (3.93-5.61)	2.04 × 10^−14^
HDL, mmol/l	1.34 (1.14-1.58)	1.29 (1.10-1.50)	1.20 (1.06-1.48)	0.322
Apolipoprotein B, mg/dl	102 (87-123)	72 (64-80)	142 (125-154)	7.88 × 10^−14^
Apolipoprotein A1, mg/dl	162 (144-184)	151 (139-176)	161 (141-181)	0.347
Lipoprotein (a), mg/dl	11.4 (5.2-33.5)	8.1 (3.4-22.8)	13.4 (6.2-33.4)	0.078
oxLDL, U/dl	68.50 (55.72-85.64)	35.29 (31.02-39.64)	118.35 (113.47-124.12)	3.15 × 10^−17^
oxLDL/LDL, U/dl per mmol per l	19.32 (16.21-23.00)	15.75 (12.64-18.53)	23.79 (20.87-28.00)	8.49 × 10^−13^
oxLDL/ApoB, U/dl per mg per dl	0.67 (0.56-0.78)	0.48 (0.42-0.55)	0.82 (0.75-1.01)	4.05 × 10^−15^
CRP, mg/dl	0.24 (0.13-0.42)	0.19 (0.09-0.37)	0.28 (0.16-0.45)	0.031
Hypertension, n (%)	586 (80.8)	39 (81.3)	37 (77.1)	0.802
Myocardial infarction, n (%)	71 (9.8)	7 (14.6)	7 (14.6)	0.772
Diabetes mellitus, n (%)	113 (15.6)	9 (18.8)	6 (12.5)	0.544
Stroke, n (%)	16 (2.2)	3 (6.3)	4 (8.3)	1.000
Statins, n (%)	163 (22.5)	19 (39.6)	13 (27.1)	0.279
Total stenosis, %	80 (25-130)	63 (23-115)	75 (25-150)	0.362
Plaque number	3 (1-4)	2 (1-4)	3 (1-5)	0.315
Mean-IMT, mm	0.71 (0.62-0.85)	0.71 (0.63-0.85)	0.71 (0.61-0.86)	0.916

BMI—body mass index, LDL—low-density lipoprotein, HDL—high-density lipoprotein, CRP—high-sensitivity C-reactive protein, oxLDL—oxidatively modified low-density lipoprotein, ApoB—Apolipoprotein B, Mean-IMT—mean intima-medial thickness. Data are presented as numbers (percentages) in cases of categorical data and median (25th–75th percentile) in cases of continuous data.

* *P*-value (difference between low and high oxLDL levels groups) for quantitative parameters was calculated for non-parametric Mann-Whitney test, for quality parameters *P*-value was calculated using two-tailed Fisher’s exact test if it is available, otherwise—for Yates’ corrected *χ*^2^ test.

The oxLDL levels varied from 21.03 to 163.72 U/dl (with the median of 68.5) and correlated with the levels of the TC, TG, LDL, CRP and ApoB, and with the ultrasound markers of atherosclerosis (S1 Table). GWAS analysis was performed for oxLDL levels and for biochemical parameters correlated with it.

**Fig 2:**
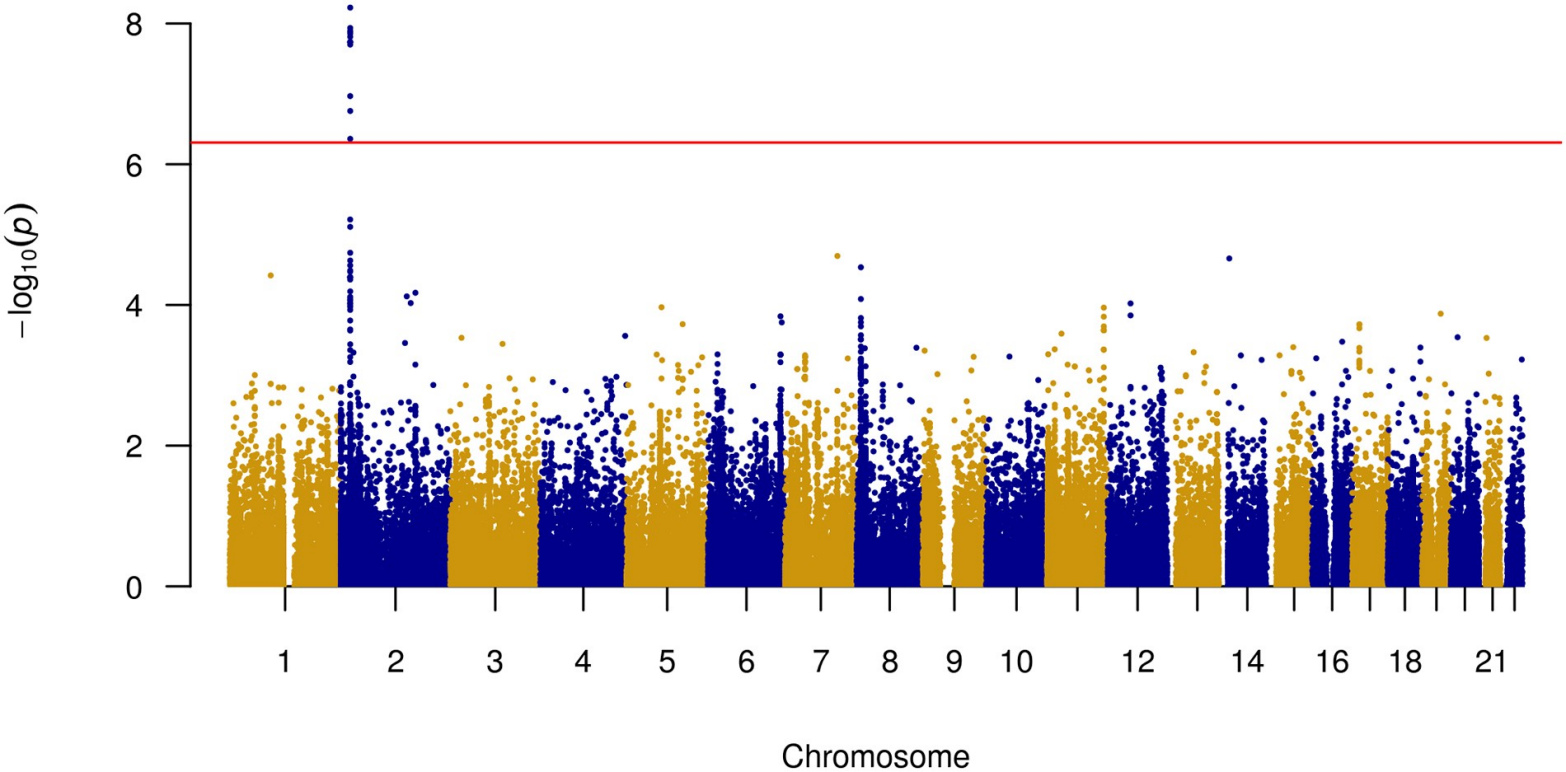
Manhattan plot of GWAS results showing highest SNPs associated with oxLDL levels. The dots represent the SNPs organized by the chromosomal order and position. The y–axis describes the statistical significance (expressed as −*log*10 of the *P*-values).

The Manhattan plot (Fig 2), quantile-quantile plot (S3 Fig) and regional plot (S4 Fig) [46] illustrate the results of the association analyses performed for the oxLDL levels. We identified 14 significant SNPs (*P* < 2.18 × 10^−7^) on the chromosome 2 (S2 Table). Twelve out of fourteen significant variants were localized in the intronic regions of *APOB* gene and in the intergenic regions near this gene. Other two SNPs were *APOB* nonsynonymous (NS) exonic variants: rs1042034 (p.Ser4338Asn) and rs676210 (p.Pro2739Leu). The association of the variants from chromosome 2 with the oxLDL/LDL and oxLDL/ApoB ratios was also found. GWAS results for other biochemical parameters were not significant in this study.

All 14 significant SNPs, found in our study and associated with the oxLDL levels, are in concordance with the variants obtained earlier in the Finnish population and replicated in the German cohorts [6]. In the latter study, only one independent SNP (rs676210) was declared as a true functional variant, which explained only 11% of the oxLDL variation. Present study showed that our significant variants explained only 6% of oxLDL variation (*R*^2^ = 6%). To this end, we suggested that the rare and low-frequency variants could be crucial for the LDL oxidation. We used the targeted sequencing of *APOB* gene locus to find other functional variants explaining an additional variation in the oxLDL levels.

### Targeted sequencing for finding variants of *APOB*

For this part of our study, we selected those 96 patients out of 725 individuals who had an extremely high (H group) and low (L group) oxLDL levels: 48 individuals with the lowest oxLDL levels (with the median of 35.29 U/dl) and 48 individuals with the highest ones (with the median of 118.35 U/dl). These groups differed significantly by oxLDL levels (*P* = 3.15 × 10^−17^ by *χ*^2^ test). H group was matched to the L group with respect to age, sex, smoking status, body mass index, waist, levels of HDL, ApoA1, and lipoprotein (a). The patients were also matched for the prevalence of hypertension, myocardial infarction, diabetes mellitus, stroke, and statin use. The severity of carotid atherosclerosis according to the results of carotid ultrasound were comparable between the study groups. Individuals from H group demonstrated significantly higher levels of TC, TG, LDL, ApoB, and CRP. The characteristics of two groups are provided in Table 1.

For targeted sequencing we designed the locus 2p24-p23 which included *APOB* gene (chr2: 20996301-21494945; GRCh37/hg19 reference human genome) and 14 significant variants according to our GWAS analysis. We conducted a targeted sequencing of designed locus and identified a total of 1,992 SNPs; 30 SNPs were exonic (Table 2) with 23 SNPs leading to the NS amino acid change including one nonsense mutation.

**Table 2:** Exonic SNPs of *APOB* found by targeted sequencing in patients with high and low oxLDL levels.

Genomic position (GRCh37/ hg19)	Exon	Amino acid change	SNP ID	SIFT/ Poly-Phen-2	MAF (ExAC)	**Low group	**High group	Previously published with respect to
21224853	29	p.Ala4481Thr	rs1801695	D / B	0.02407	47,1,0	48,0,0	HDL [47]; oxLDL [6, 28]; Dementia [48]
21224854*	29	p.Gln4480Gln			0.000008267	47,1,0	48,0,0	No
21224907	29	p.Lys4463Glu		D / B		48,0,0	47,1,0	No
21225119	29	p.Ser4392Asn		T / B	0.00004974	48,0,0	47,1,0	Familial hypercholesterolemia [49]
21225281	29	p.Ser4338Asn	rs1042034	T / B	0.7057	7,24,17	1,6,41	HDL, TG [50]; LDL [51–53]; Ischemic Stroke [54]; Hyperlipidemia [55]; oxLDL [6]; TC [52, 53, 56, 57]
21225485	29	p.Arg4270Thr	rs1801702	T / B	0.0456	46,2,0	45,3,0	TC, LDL [58]
21225500	29	p.Val4265Ala	rs61743502	T / B	0.005139	48,0,0	47,1,0	Familial hypercholesterolemia [59]
21225753	29	p.Glu4181Lys	rs1042031	T / B	0.153	41,7,0	37,9,2	ADH [60]; Carotid plaques [61]; Hipertension, TG [62]; Serum lipid levels [63]; Familial hypercholesterolemia [64, 65]; HDL [58]; HDL, LDL [66]; Breast Cancer [67]; Ischemic Stroke [68]
21225912	29	p.Val4128Met	rs1801703	T / B	0.006171	46,2,0	48,0,0	Exceptional longevity [69]
21228339	26	p.Ser3801Thr	rs12713540	T / P	0.001046	48,0,0	47,1,0	PDR [70]
21228827	26	p.Arg3638Gln	rs1801701	T / B	0.06887	43,5,0	42,6,0	LDL [51]; TG [63]; CAD [71]; Familial hypercholesterolemia [64]; Ischemic Stroke [68]
21229446	26	p.Gln3432Glu	rs1042023	T / B	0.007588	47,1,0	48,0,0	LDL receptor binding [72, 73]; Familial hypercholesterolemia [74]
21229609	26	p.Leu3377Leu	rs1799812		0.006083	46,2,0	48,0,0	ADH [60]
21231524	26	p.Pro2739Leu	rs676210	D / D	0.2928	17,24,7	41,6,1	MI [75]; oxLDL [6, 28]; TC [56]; Lipid metabolism phenotypes [76]; Familial hypercholesterolemia [64]
21231592	26	p.Ile2716Ile	rs6413458		0.01952	47,1,0	45,3,0	Lp-PLA2 [77]; ADH [60]
21232125*	26	p.Val2539Ile	rs148170480	T / B	0.002399	47,1,0	48,0,0	No
21232128*	26	p.Leu2538Leu	rs72653093		0.001509	48,0,0	46,2,0	No
21232195	26	p.Thr2515Thr	rs693		0.3899	17,24,7	7,22,19	Ischemic Stroke [54]; LDL [78–81]; Carotid plaques [61]; TC [79]; TG [80]; Breast Cancer [67]
21232341	26	p.Lys2467Ter		T / -		48,0,0	47,1,0	No
21233877*	26	p.Val1955Met	rs368970025	T / B	0.00008238	47,1,0	48,0,0	No
21233972*	26	p.His1923Arg	rs533617	D / D	0.03116	45,3,0	48,0,0	LDL, TC [82, 83]; oxLDL [6, 28]
21234915	26	p.Leu1609Leu	rs72653083		0.001726	47,1,0	48,0,0	No
21236221	25	p.Pro1343Ser	rs374427541	D / D	0.00003295	47,1,0	48,0,0	No
21238367*	22	p.Arg1128His	rs12713843	D / B	0.003708	47,0,1	48,0,0	LDL, TC [84]
21238413*	22	p.Asp1113His	rs12713844	D / P	0.006858	48,0,0	47,1,0	Spastic Paraplegia [85]; Familial hypercholesterolemia [86]
21245813*	18	p.Asn902Asn	rs1801700		0.03445	46,2,0	39,9,0	ADH [60]
21245889*	18	p.Pro877Leu	rs12714097	D / D	0.000479	48,0,0	47,1,0	Familial hypercholesterolemia [87]
21249716	15	p.Val730Ile	rs12691202	T / B	0.02504	47,1,0	44,4,0	ADH [60]
21250914	14	p.Ala618Val	rs679899	T / D	0.4857	19,16,13	30,15,3	FH [65]; MI [75]; LDL [51]; Hyperlipidemia [55]; Chronic kidney disease [88]; Coronary heart disease [89]; oxLDL [6, 28]
21263900	4	p.Thr98Ile	rs1367117	T / B	0.2619	29,14,5	18,24,6	LDL, TC [50, 90]; Lipid metabolism phenotypes [76]

SIFT score: T—Tolerated, D—Deleterious; PolyPhen-2 score: B—Benign, P—Possibly damaging, D—Probably damaging; ADH—autosomal dominant hypercholesterolemia, CAD—coronary artery disease, HDL—high density lipoprotein, LDL—low density lipoprotein, Lp-PLA2—lipoprotein-associated phospholipase A2, MI—myocardial infarction, oxLDL—oxidatively modified low density lipoprotein, PDR—proliferative diabetic retinopathy, TC—total cholesterol, TG—triglycerides.

*SNPs significantly associated with oxLDL levels by BE-SKAT;

**Number of patients with regarded variants genotypes presented as homozygous reference allele, heterozygous, homozygous alternative allele.

Eleven variants were present only in the L group. Six of them were rare NS variants: rs1801703 (p.Val4128Met), rs1042023 (p.Gln3432Glu), rs148170480 (p.Val2539Ile), rs368970025 (p.Val1955Met), rs374427541 (p.Pro1343Ser), rs12713843 (p.Arg1128His). Two NS variants unique for the L group were not singletons, they were found more than in one patient. Rare NS variant rs1801703 (p.Val4128Met) was found in two individuals, and the NS low-frequency SNP rs533617 (p.His1923Arg) was presented in three individuals. Moreover, two patients from the L group had synonymous change rs1799812 (p.Leu3377Leu) not presented in the H group.

Eight SNPs were unique to the H group. All NS variants out of them were rare singletons: five previously reported (p.Ser4392Asn, rs61743502 (p.Val4265Ala), rs12713540 (p.Ser3801Thr), rs12713844 (p.Asp1113His), rs12714097 (p.Pro877Leu)) and two novel variants (p.Lys4463Glu and p.Lys2467Ter). Also, two patients from the H group had synonymous change rs72653093 (p.Leu2538Leu) presented only in this group of patients.

For each variant out of 30 exonic variants obtained by the targeted sequencing, the MAF was received from the Exome Aggregation Consortium (ExAC) database (http://exac.broadinstitute.org/). There were two novel variants with no MAF data in our SNPs set. In order to account for these variants in the variant association testing, their MAF was defined as the minimal MAF observed in our SNPs set of 30 exonic variants.

Additionally, to exclude linked variants we performed linkage disequilibrium (LD) analysis based on 1000 Genomes Project data (S5 Fig). Two common variants rs676210 and rs1042034 in the considered SNPs set were in a perfect LD according to the public data; moreover, they were in a perfect LD according to our study. We excluded rs1042034 variant. The other four variants (rs6413458 and rs1801702, rs1799812 and rs1801703), which are in LD according to 1000 Genomes Project data, had the low MAF and were not linked in our study. We did not exclude any of these variants. Thus, we excluded only one variant rs1042034 and for the further variant association testing, we used 29 out of 30 exonic SNPs.

### Variant association testing

Initially, to find the association between the genetic variants and the oxLDL levels we used the WST [18], SKAT (with/without adjustment for covariates) [19] and optimal SKAT (SKAT-O) method which is based on combination of burden test and SKAT (with/without adjustment for covariates) [20]. As covariates we used age, sex, smoking status, body mass index, waist, HDL, CRP, lipoprotein (a), hypertension, myocardial infarction, diabetes mellitus, stroke and statins use.

First of all, we analyzed the rare SNPs out of 29 variants. We considered (i) the subgroup of variants defined as deleterious/damaging (DD) by the SIFT and PolyPhen-2 algorithms as the most plausible functional variants, (ii) the subgroup of NS variants, (iii) the entire group of rare variants (with synonymous and NS amino acid change). The same testing steps were conducted for the rare and low-frequency variants together. The results of the analysis are presented in Table 3.

**Table 3:** Association analysis between exonic *APOB* variants and oxLDL levels.

	Rare variants (MAF<0.01)					Rare and low-frequency variants (MAF<0.05)				
Test	WST	SKAT	SKAT with covariates	SKAT-O	SKAT-O with covariates	WST	SKAT	SKAT with covariates	SKAT-O	SKAT-O with covariates
Deleterious/damaging*	0.244	0.464	0.358	0.645	0.443	0.623	**0.037**	**0.004**	**0.043**	**0.006**
Nonsynonymous	0.829	0.164	**0.048**	0.253	0.089	0.813	0.164	**0.034**	0.278	0.065
Synonymous & nonsynonymous	0.789	0.087	**0.034**	0.126	0.064	0.173	**0.017**	**0.003**	**0.034**	**0.006**

*By SIFT and PolyPhen-2 algorithms.

As follows from Table 3, the WST did not overcome the required level of significance in none of the groups, which could be due to the large number of multidirectional and neutral variants. However, the SKAT-O adjusted for covariates did not overcome the level of significance in the group of rare variants as well. The SKAT-O analysis showed that the best combination of the burden test and SKAT method was the SKAT test itself. The behavior of SKAT-O depending on the combination coefficient is shown on S6 Fig. Thus, in the following analysis, we used only the SKAT, because the corresponding *P*-value was calculated more accurately. As it can be seen in Table 3, the addition of covariates decreased the *P*-value.

Testing with the SKAT adjusted for the covariates showed no association between the rare DD variants and the oxLDL levels (*P* = 0.358). The *P*-value of the former with addition of the low-frequency DD variant rs533617 (p.His1923Arg) decreased to *P* = 0.004. The functional prediction algorithms could fail the correct identification of the any NS sequence variations, as it was shown for the SNPs associated with the LDL and the dominant hypercholesterolemia [91]. Therefore, the consideration of other variants seemed reasonable.

The NS variants that alter an amino acid can change the protein function [38], thus such variants are more likely to affect the phenotype compared to the synonymous variants. However, even if the SNP does not change amino acid, it can still affect the gene function by altering the mRNA stability or splicing [92, 93]. The analysis of the NS variants subgroup and of the subgroup with the synonymous and the NS variants showed a significant association with the oxLDL levels according to SKAT adjusted for covariates. The *P*-value was the least in the subgroup with the synonymous and the NS variants. This indicates that both NS SNPs and synonymous contribute to the oxLDL levels. Additionally, the *P*-value decreased in all subgroups of the rare variants after adding the variants with the higher MAF—low-frequency variants.

**Fig 3:**
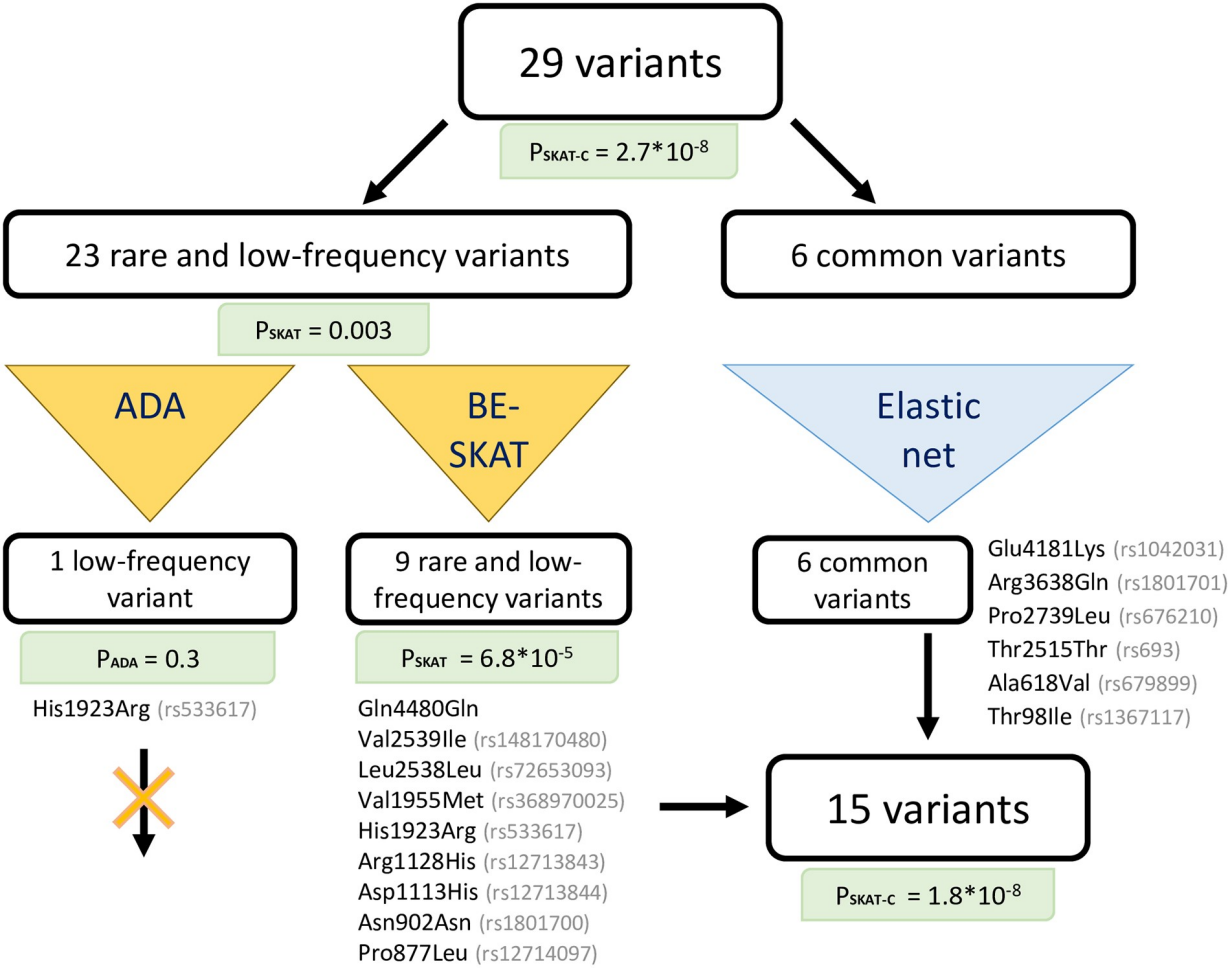
Joint analysis of rare, low-frequency and common exonic variants obtained by targeted sequencing.

Then, we conducted the joint analysis of all 29 variants which included rare, low-frequency and common variants. For this purpose, we used the SKAT-C method which showed the significant association of all 29 SNPs with oxLDL levels (*P* = 2.7 × 10^−8^). However, some of these variants could be neutral, so they should be excluded from the analysis. Methods for finding the causal variants differ depending on variants frequency. Therefore, we divided the SNPs set of 29 variants into two subgroups: rare with low-frequency variants and common variants. The scheme of the joint testing of these SNPs is shown on the Fig 3.

For the subgroup of rare and low-frequency variants we applied the ADA [24] and BE-SKAT [21] tests. ADA test chose the variant rs533617 (p.His1923Arg) with the *P* = 0.3. We used ADA implementation based on the Fisher’s test. We presume that the small size of patients group caused the high *P*-values in the univariate analysis by the Fisher’s test, so this could cause the failure of the ADA test. As noted in [23], the BE-SKAT is less conservative than the ADA test, i.e. this method can select more variants. However, the BE-SKAT can leave more neutral variants than ADA. The BE-SKAT selected 9 variants: p.Gln4480Gln, rs148170480 (p.Val2539Ile), rs72653093 (p.Leu2538Leu), rs368970025 (p.Val1955Met), rs533617 (p.His1923Arg), rs12713843 (p.Arg1128His), rs12713844 (p.Asp1113His), rs1801700 (p.Asn902Asn), rs12714097 (p.Pro877Leu). The initial *P*-value of the SKAT was reduced from 0.003 in 23 variants group to 6.8 × 10^−5^ in 9 variants group.

For the subgroup of common variants, we applied an elastic net with cross-validation based on the AUC-ROC metric [44]. According to the present analysis, all considered common variants were used in the model. Thereby, we included all 6 common variants in further analysis.

Finally, we combined the analysis results of both subgroups (rare and low-frequency variants and common variants) and revealed a set of 15 variants for which the SKAT-C method rejected the null hypothesis of the absence of association at a required significance level (*P* = 1.8 × 10^−8^). Thus, as the result of our statistical analysis, predominantly neutral variants were excluded from the analysis, reducing the number of variants and the *P*-value.

Six common variants included in the set of 15 SNPs, associated with oxLDL levels by the SKAT-C, previously were associated with the LDL, TG and other lipid parameters and different cardiovascular events (see Table 2). They may affect the oxLDL levels but significant associations were confirmed in the GWAS separately only for the rs676210 variant.

Rare and low-frequency variants were more interesting in the context of finding the ‘missing heritability’ and explaining the variation in oxLDL levels. The SNP rs533617 (p.His1923Arg) was a potentially causal variant by the statistical analysis. It was presented only in three individuals from the L group and was predicted to be the DD. However, this variant was observed only in patients with the SNP rs676210 significantly associated with the oxLDL by GWAS. For this reason, we could neither confirm nor entirely exclude the contribution of variant rs533617 to the decreased oxLDL levels.

The synonymous variant rs72653093 (p.Leu2538Leu) was presented in two individuals from the H group. It was included in the SNPs set obtained by BE-SKAT and was not published previously. The other non singleton variants rs1801703 (p.Val4128Met) and rs1799812 (p.Leu3377Leu), found only in the L group, were excluded by the BE-SKAT as neutral variants. This could be due to the covariates adjustment or due to the small sample size.

The single patient in the L group was homozygous for variant rs12713843 (p.Arg1128His). This SNP was predicted to be deleterious by the SIFT but benign by PolyPhen-2. This difference may be due to the difference in mathematical algorithms underlying methods. Since SIFT and PolyPhen-2 algorithms do not provide the evidence that this SNP might be the deleterious one for sure, their predictions should be interpreted with caution. Previously the variant rs12713843 was associated with lower LDL and lower TC levels [84]. The carrier of this SNP had lower levels of these parameters in comparison to the median value in the L group (TC = 3.76 mmol/l, LDL = 1.29 mmol/l, both lower than the 25th percentile) and the minimal oxLDL level among the entire cohort (oxLDL = 21.03 U/dl). Thus, the rs12713843 variant may be causative variant for decreasing oxLDL level. However, this decrease may not be caused by the oxidation, but it is due to the reduced LDL and TC levels, described in [84]. Additionally, the carrier of this SNP had the other variant rs676210, associated with the decreased oxLDL level.

NS singletons rs12713844 (p.Asp1113His) and rs12714097 (p.Pro877Leu) presented in the H group were selected by the BE-SKAT. Both were predicted to be deleterious and both previously reported with respect to the familial hypercholesterolemia (Table 2). Novel variants p.Lys4463Glu and p.Lys2467Ter, presented only in the H group, were excluded by the BE-SKAT as being neutral.

### *APOB* gene evolutionary conservation analysis

**Fig 4:**
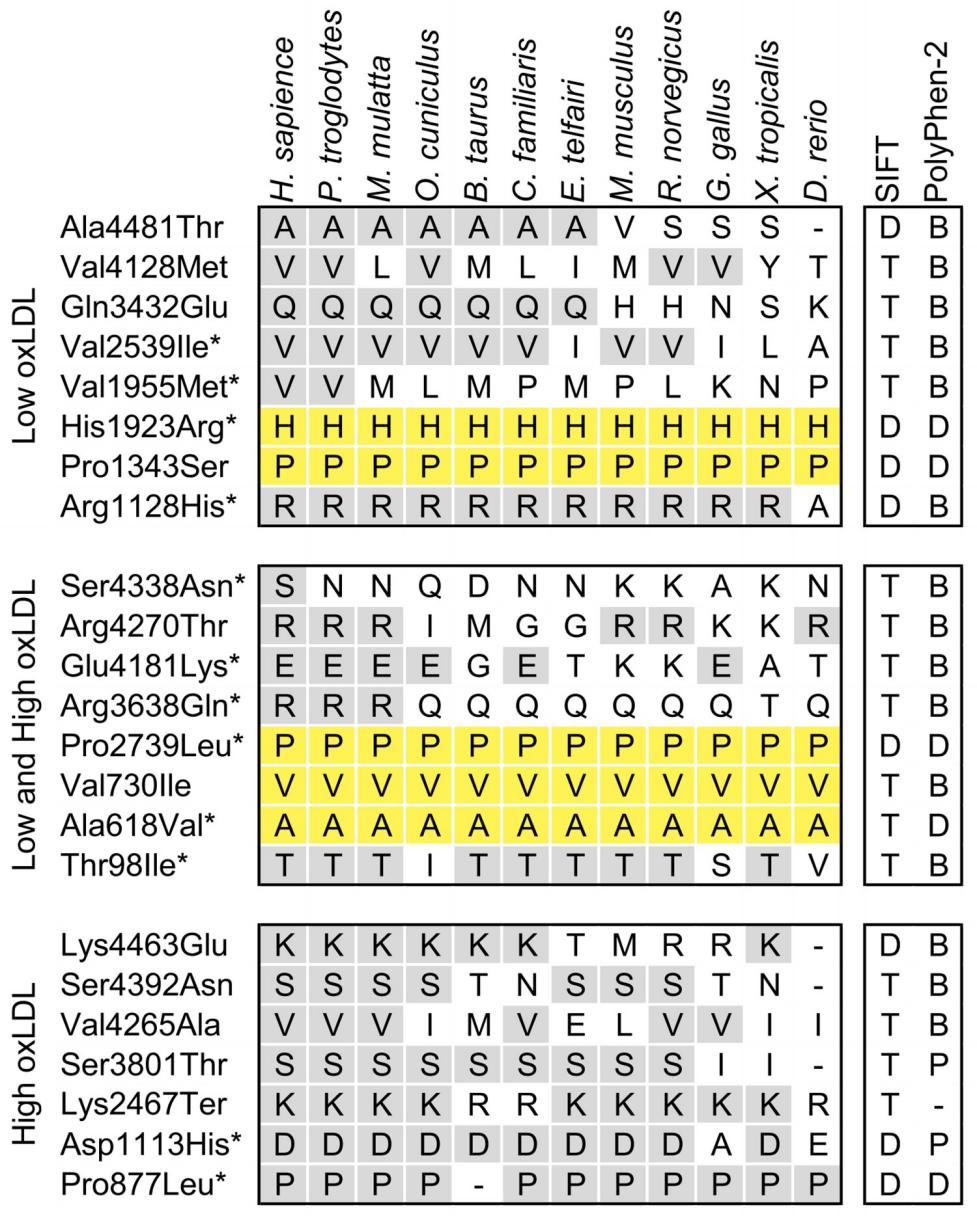
Evolutionary sequence conservation and predicted functional effects of nonsynonymous sequence variations in *APOB*. Conservation for each variation found in the low oxLDL subjects only (top), the both low and high oxLDL subjects (middle), or high oxLDL levels group (bottom). An asterisk (*) indicates the SNPs significantly associated with oxLDL levels by the BE-SKAT. The alignment includes *Homo sapiens* (human), *Pan troglodytes* (chimpanzee), *Macaca mulatta* (Rhesus monkey), *Oryctolagus cuniculus* (rabbit), *Bos taurus* (cow), *Canis lupus familiaris* (dog), *Echinops telfairi* (hedgehog), *Mus musculus* (house mouse), *Rattus norvegicus* (rat), *Gallus gallus* (chicken), *Xenopus tropicalis* (frog), *Danio rerio* (zebrafish). The predicted effect of each variant on protein function is indicated (right columns). SIFT: T—tolerated; D—deleterious. PolyPhen-2: B—benign; P—possibly damaging; D—probably damaging.

To examine the relationship between the evolutionary conservation and functional effects of the NS sequence variations, identified by targeted sequencing, we aligned the human ApoB amino acid sequence with the sequences from 11 other species (Fig 4).

Two SNPs rs533617 (p.His1923Arg) and rs374427541 (p.Pro1343Ser) in the L group were highly conserved from human to zebrafish and one (rs12713843 (p.Arg1128His)) to frog. There were three substituted amino acids conserved in primates and some other mammals: rs1801695 (p.Ala4481Thr), rs1042023 (p.Gln3432Glu), rs148170480 (p.Val2539Ile).

In the H group two changed amino acid residues rs12713540 (p.Ser3801Thr) and rs12713844 (p.Asp1113His) were completely conserved from primates to rats, and rs12714097 (p.Pro877Leu) was conserved from human to zebrafish, except cow.

Both groups included three common variants highly conserved from human to zebrafish: rs676210 (p.Pro2739Leu), rs12691202 (p.Val730Ile) and rs679899 (p.Ala618Val).

It was initially assumed that highly conserved NS variation at amino acid residues would be found only in either L or H group, as demonstrated previously for the genetic variation in *NPC1L1* [15]. However, we found several variants common to both extremes. Also, it was shown previously for the variants of *PCSK9* [91] and *APOB* [51].

It is also interesting that two SNPs from the L group were highly conserved in all regarded species and had the DD status. While we suggested that the low oxLDL level is a healthier trait in comparison with the increased oxLDL, high evolutionary sequence conservation suggests that the variants, responsible for the lowering of the oxLDL levels, also cause the loss of any other important functions. Thus, the positive effect of variants which decrease oxLDL level and deleterious effect revealed by *in silico* prediction methods remain controversial.

## Conclusion

The major finding of this study is association of the oxLDL levels with both common and rare variants of the *APOB* gene. For the first time, we showed the association of the rare variants with the circulating oxLDL levels. Additionally, we performed the joint analysis of rare, low-frequency and common *APOB* variants. Whereas the group of variants associated with oxLDL levels was revealed, the rare or novel variants still should be interpreted cautiously. It is necessary to evaluate the variant and the gene in the context of the patient’s and family’s history, physical examinations, and previous laboratory tests to distinguish between variants that cause the patient’s disorder and those that are benign. Functional evaluation can also provide a more definitive assessment of the variant pathogenicity.

*APOB* gene variants can change secondary structure of the human ApoB and the LDL particle size in comparison with the wild type LDL particle [94]. These changes may also impact the LDL oxidation. To determine the molecular biological effects of the genetic variant as well as the interactions with other variants and to understand the mechanisms of oxidation, mathematical modeling and high-throughput technologies such as mass spectrometry or infrared spectroscopy analysis could be applied. Such studies of *APOB* variants may provide useful information to understand their role in the LDL oxidation and atherosclerosis progression.

## Supporting information

S1 Fig Binding specificity of native low density lipoprotein (nLDL), malondialdehyde-modified LDL (MDA-LDL), glyoxal-modified LDL (G-LDL) and methylglyoxal-modified LDL (MG-LDL) to the mAb-4E6 antibody. (TIF)

S2 Fig Principal component analysis (PCA) for GWAS samples. A. The colors ranged from red (high oxLDL levels) to blue (low oxLDL levels). The matching suggested minimal evidence of population stratification. B. The colors indicate 48 individuals with the highest oxLDL levels (red) and 48 individuals with the lowest oxLDL levels (blue).
(TIFF)

S3 Fig Quantile-quantile plot of the observed and expected *P*-values obtained from GWAS with oxLDL levels showing a clear deviation from normal distribution. (TIF)

S4 Fig Regional plot of the *APOB* region with SNPs significantly associated with oxLDL levels. Dots show *P*-values from GWAS on the −*log*10 scale (vertical axis), and the chromosomal position (horizontal axis). Pairwise linkage disequilibrium (*r*^2^) from the most significant SNP (rs11902417) is color-coded. The light blue curve shows the local recombination rate based on 1000 Genomes EUR data (release Nov 24, 2014).
(TIF)

S5 Fig Heatmap of pairwise linkage disequilibrium (LD) statistics for *APOB* gene exonic variants. The colors of the cells refer to r^2^ values and show the amount of LD between two markers. LD statistics between a pair of SNPs was calculated using haplotype CEU data from the 1000 Genomes Project.
(TIF)

S6 Fig P-value of SKAT-O with covariates on synonymous and nonsynonymous variants. The plot illustrates the behavior of SKAT-O and its dependence on the combination coefficient of SKAT and Burden test. If the coefficient is equal to null, SKAT-O behaves as SKAT. If the coefficient is equal to one, SKAT-O behaves as Burden test.
(TIF)

S1 Dataset Capture design coordinates for targeted sequencing. TargetSeq Custom Enrichment Kit was designed to target the region containing the complete genomic sequence of the *APOB* gene in locus 2p24-p23 (chr2: 20996301-21494945; GRCh37/hg19 reference human genome).
(DOC)

S1 Appendix Materials and methods for more details. (PDF)

S1 Table Correlation of oxLDL levels with biochemical parameters and ultrasound markers of atherosclerosis. (PDF)

S2 Table Fourteen the most significant SNPs obtained by GWAS for oxLDL levels in 725 samples. All SNPs are localized on Chromosome 2 in *APOB* gene region.
(PDF)
