# Supplementary figures and images for: Multiple rare and common variants in APOB gene locus associated with oxidatively modified low-density lipoprotein levels
Source: PLoS One. 2019 May 31;14(5):e0217620. doi: 10.1371/journal.pone.0217620 (PMC6544350; doi:10.1371/journal.pone.0217620)

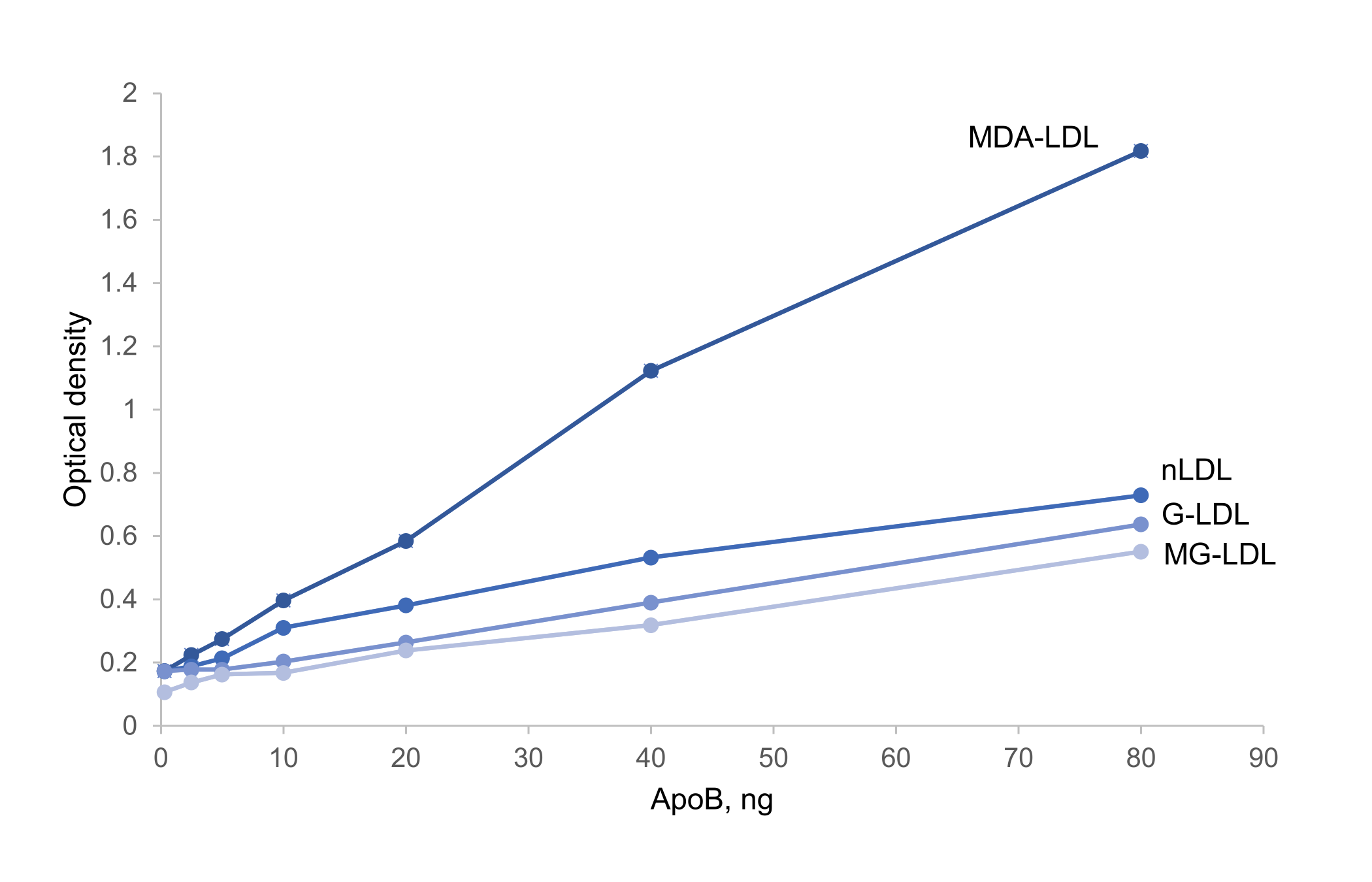

Supplement: S1 Fig — (TIF) [file pone.0217620.s001.tif]

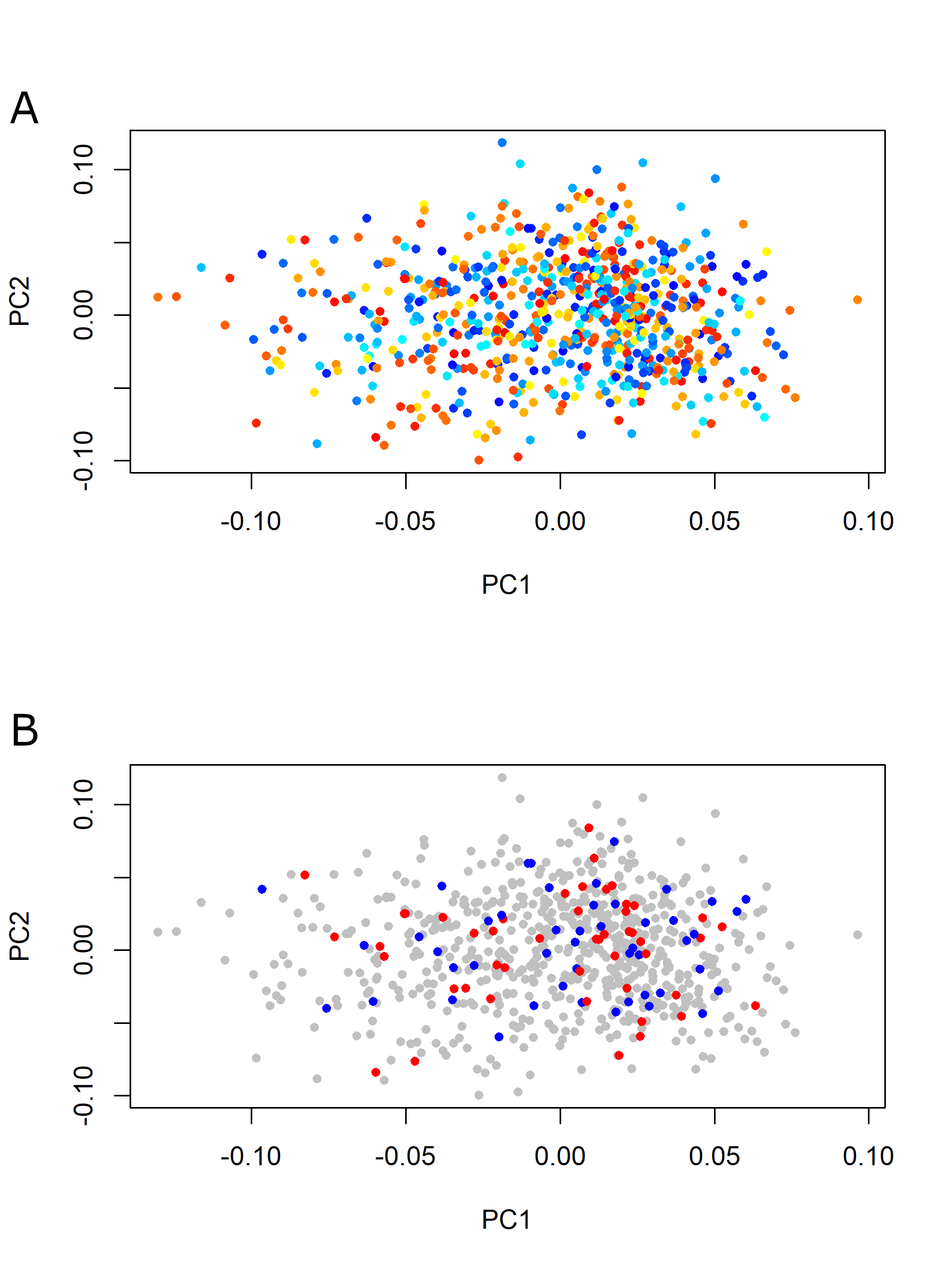

Supplement: S2 Fig — A. The colors ranged from red (high oxLDL levels) to blue (low oxLDL levels). The matching suggested minimal evidence of population stratification. B. The colors indicate 48 individuals with the highest oxLDL levels (red) and 48 individuals with the lowest oxLDL levels (blue). (TIFF) [file pone.0217620.s002.tiff]

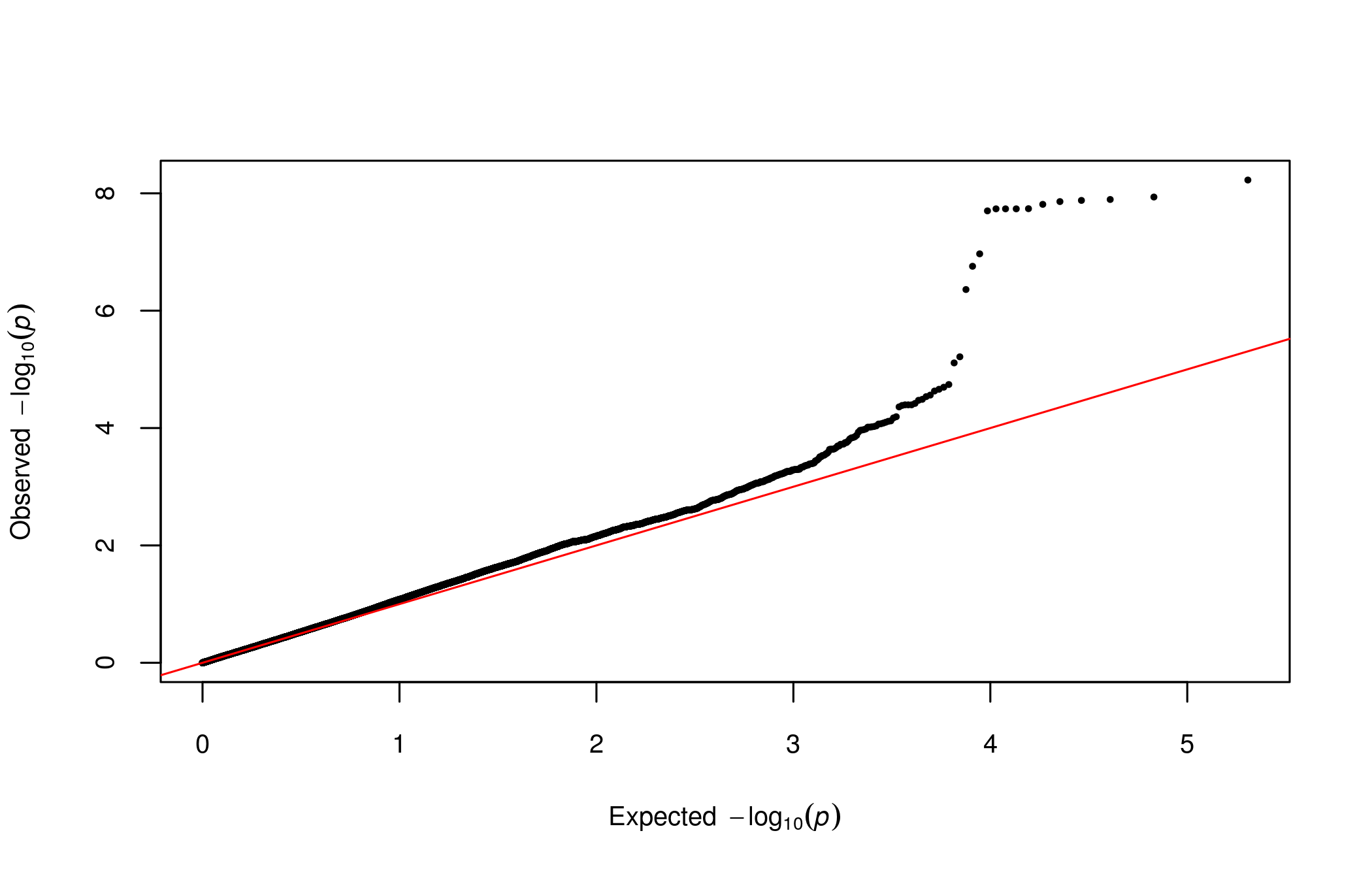

Supplement: S3 Fig — (TIF) [file pone.0217620.s003.tif]

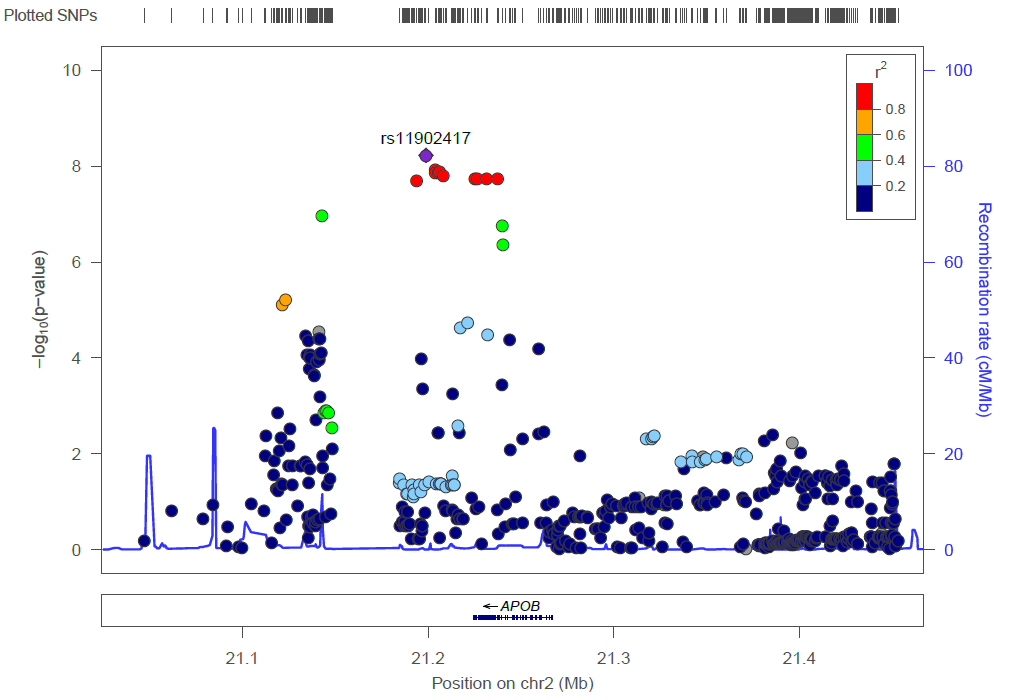

Supplement: S4 Fig — Dots show P-values from GWAS on the −log10 scale (vertical axis), and the chromosomal position (horizontal axis). Pairwise linkage disequilibrium (r2) from the most significant SNP (rs11902417) is color-coded. The light blue curve shows the local recombination rate based on 1000 Genomes EUR data (release Nov 24, 2014). (TIF) [file pone.0217620.s004.tif]

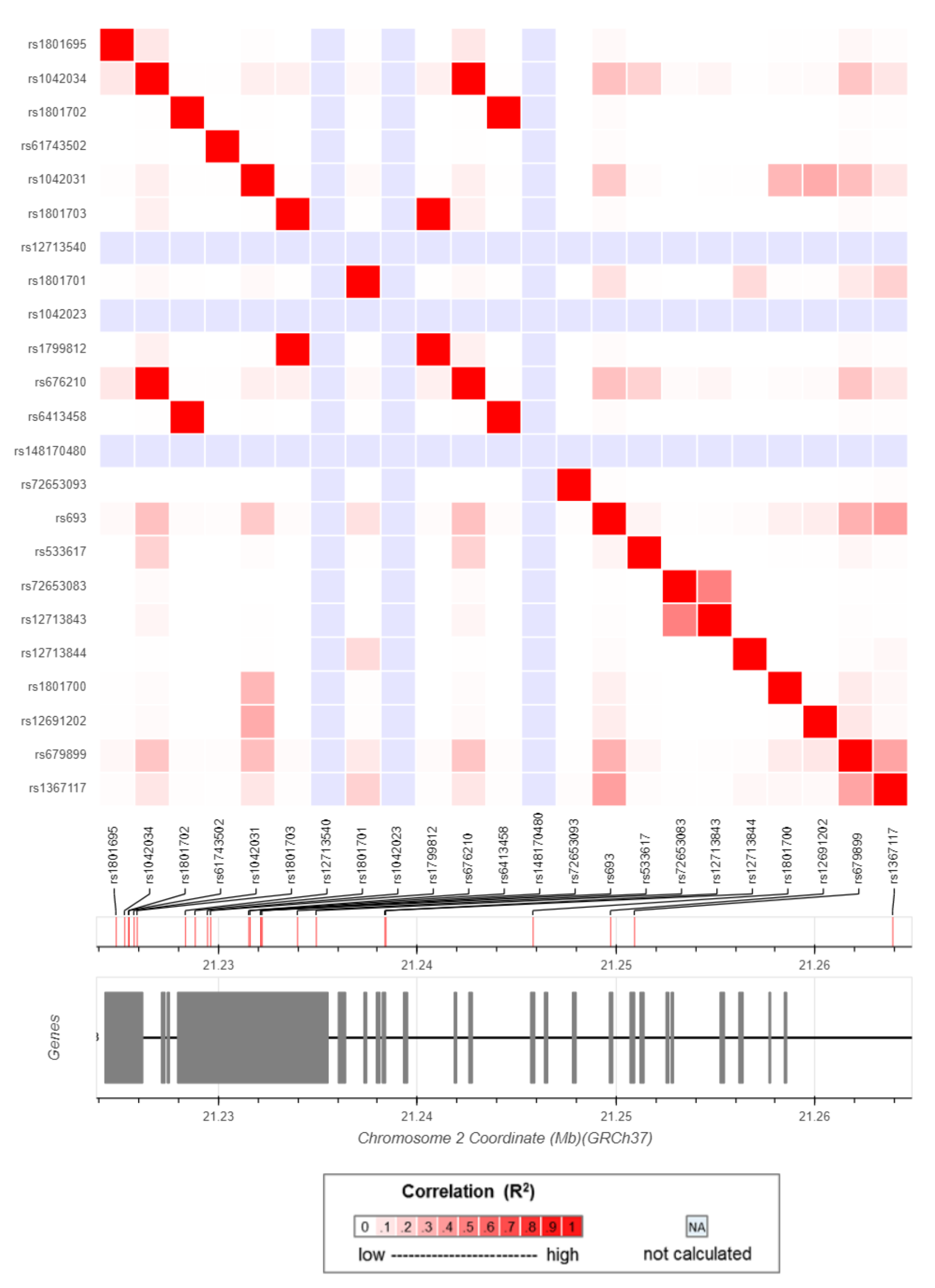

Supplement: S5 Fig — The colors of the cells refer to r2 values and show the amount of LD between two markers. LD statistics between a pair of SNPs was calculated using haplotype CEU data from the 1000 Genomes Project. (TIF) [file pone.0217620.s005.tif]

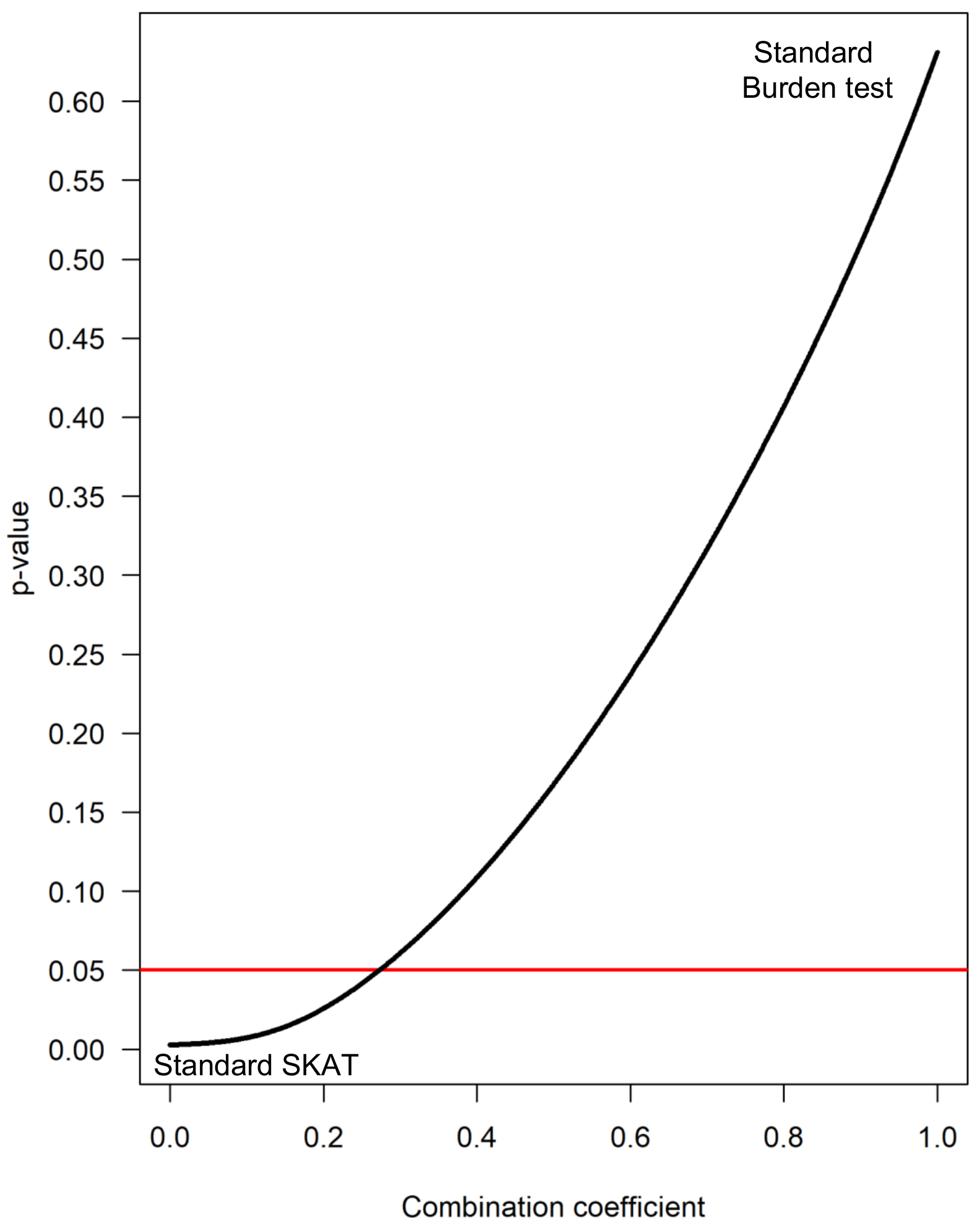

Supplement: S6 Fig — The plot illustrates the behavior of SKAT-O and its dependence on the combination coefficient of SKAT and Burden test. If the coefficient is equal to null, SKAT-O behaves as SKAT. If the coefficient is equal to one, SKAT-O behaves as Burden test. (TIF) [file pone.0217620.s006.tif]
