# Supplementary material for: Multiple rare and common variants in APOB gene locus associated with oxidatively modified low-density lipoprotein levels
Source: PLoS One. 2019 May 31;14(5):e0217620. doi: 10.1371/journal.pone.0217620 (PMC6544350; doi:10.1371/journal.pone.0217620)
Supplement: S1 Dataset — TargetSeq Custom Enrichment Kit was designed to target the region containing the complete genomic sequence of the APOB gene in locus 2p24-p23 (chr2: 20996301-21494945; GRCh37/hg19 reference human genome). (DOC) [file pone.0217620.s007.doc]

TargetSeq Custom Enrichment Kit was designed to target the region containing the complete genomic sequence of the *APOB* gene in locus 2p24-p23 (chr2: 20996301-21494945; GRCh37/hg19 reference human genome). The kit consists of 536 fragments accounting for a total of 391 833 bp.

Capture design coordinates:

track name=tiled_region description="Tiled Regions"

chr2 20996273 20997531

chr2 20997593 20998255

chr2 20998258 20998333

chr2 20998363 20998534

chr2 20998643 20998710

chr2 20999003 20999099

chr2 20999153 21000358

chr2 21000678 21000852

chr2 21000858 21001698

chr2 21002103 21002917

chr2 21002918 21003691

chr2 21003728 21004250

chr2 21004258 21005178

chr2 21005188 21005654

chr2 21005673 21006645

chr2 21006678 21006926

chr2 21006948 21007088

chr2 21007098 21007434

chr2 21007453 21007683

chr2 21007703 21007859

chr2 21007868 21008063

chr2 21008073 21008144

chr2 21008438 21008883

chr2 21009173 21009280

chr2 21009343 21010537

chr2 21010683 21010752

chr2 21011058 21011239

chr2 21011533 21012836

chr2 21012868 21013558

chr2 21013593 21014112

chr2 21014113 21014207

chr2 21014428 21014876

chr2 21014913 21015150

chr2 21015158 21015227

chr2 21015263 21015330

chr2 21015358 21015736

chr2 21015738 21016528

chr2 21016533 21016605

chr2 21016613 21016948

chr2 21016983 21017245

chr2 21017283 21018318

chr2 21018333 21019217

chr2 21021543 21021897

chr2 21022188 21023376

chr2 21023688 21024024

chr2 21024053 21024664

chr2 21024668 21024741

chr2 21024773 21024849

chr2 21024883 21025282

chr2 21025303 21025430

chr2 21025433 21025959

chr2 21026008 21026295

chr2 21026393 21026708

chr2 21026773 21026845

chr2 21027593 21028949

chr2 21029388 21029521

chr2 21029538 21029612

chr2 21029978 21030176

chr2 21030453 21032022

chr2 21032023 21032190

chr2 21032193 21032369

chr2 21032378 21032686

chr2 21032718 21033092

chr2 21033113 21033665

chr2 21033808 21034225

chr2 21034243 21034337

chr2 21034538 21035341

chr2 21035368 21035953

chr2 21035968 21036031

chr2 21036288 21036704

chr2 21036778 21037191

chr2 21037473 21037582

chr2 21037588 21038810

chr2 21038848 21038967

chr2 21039133 21039268

chr2 21039283 21039361

chr2 21040188 21040295

chr2 21040393 21041619

chr2 21041658 21042748

chr2 21043038 21043844

chr2 21043848 21043928

chr2 21043933 21046031

chr2 21046248 21046390

chr2 21046403 21048362

chr2 21048373 21048547

chr2 21048613 21049095

chr2 21049363 21050198

chr2 21050203 21050420

chr2 21050738 21050933

chr2 21050938 21051592

chr2 21051613 21051821

chr2 21052118 21053282

chr2 21053308 21053593

chr2 21053663 21055113

chr2 21055128 21055238

chr2 21055363 21056760

chr2 21056768 21058026

chr2 21058043 21058388

chr2 21058403 21060063

chr2 21060073 21062231

chr2 21062338 21062442

chr2 21062453 21062593

chr2 21062678 21062782

chr2 21062793 21063553

chr2 21063753 21063944

chr2 21064533 21064633

chr2 21064833 21064907

chr2 21065448 21065752

chr2 21065788 21067094

chr2 21067423 21067746

chr2 21067773 21067845

chr2 21067913 21067985

chr2 21068128 21068755

chr2 21068888 21069106

chr2 21069243 21069410

chr2 21069413 21069485

chr2 21069508 21069657

chr2 21069693 21069769

chr2 21069828 21070636

chr2 21070673 21071508

chr2 21071523 21071827

chr2 21075793 21076254

chr2 21076258 21078240

chr2 21078383 21078480

chr2 21078628 21078697

chr2 21078698 21079119

chr2 21079183 21079292

chr2 21079643 21081374

chr2 21081403 21081516

chr2 21081598 21082712

chr2 21082713 21083558

chr2 21083638 21084542

chr2 21085248 21086085

chr2 21086123 21087131

chr2 21087238 21087354

chr2 21087513 21087657

chr2 21088118 21088260

chr2 21088263 21088614

chr2 21088628 21088833

chr2 21089148 21089248

chr2 21089258 21089501

chr2 21089788 21089966

chr2 21090043 21090143

chr2 21090228 21090478

chr2 21090483 21090651

chr2 21090743 21090883

chr2 21090893 21090966

chr2 21090973 21091101

chr2 21091118 21091359

chr2 21091428 21091533

chr2 21091558 21092522

chr2 21092638 21092716

chr2 21092718 21092871

chr2 21092898 21093001

chr2 21093068 21093137

chr2 21093213 21093287

chr2 21094043 21094117

chr2 21094128 21094202

chr2 21094203 21094526

chr2 21094783 21095298

chr2 21095618 21095944

chr2 21095973 21096361

chr2 21096668 21097152

chr2 21097298 21098869

chr2 21099228 21100804

chr2 21101108 21103776

chr2 21103798 21104086

chr2 21104613 21104748

chr2 21104778 21105221

chr2 21111358 21111690

chr2 21111703 21112786

chr2 21112838 21113360

chr2 21113383 21120220

chr2 21120253 21120920

chr2 21120933 21121184

chr2 21121243 21121374

chr2 21121378 21121522

chr2 21121528 21121631

chr2 21121643 21121917

chr2 21121933 21123508

chr2 21123518 21126626

chr2 21126638 21127347

chr2 21127458 21127600

chr2 21127993 21128077

chr2 21128083 21128165

chr2 21129143 21129212

chr2 21129508 21129718

chr2 21129728 21129801

chr2 21130033 21130146

chr2 21130158 21136112

chr2 21136118 21136813

chr2 21136843 21139253

chr2 21139293 21140459

chr2 21140463 21140947

chr2 21140948 21141610

chr2 21141623 21143256

chr2 21143268 21143396

chr2 21143753 21145588

chr2 21145593 21146010

chr2 21146133 21146314

chr2 21146348 21146585

chr2 21146598 21146673

chr2 21146783 21147817

chr2 21147823 21151754

chr2 21151803 21152546

chr2 21152563 21153113

chr2 21178113 21179670

chr2 21179778 21183656

chr2 21183713 21183792

chr2 21183858 21183936

chr2 21184053 21186541

chr2 21186563 21186808

chr2 21186818 21187732

chr2 21187753 21189274

chr2 21189283 21190690

chr2 21190993 21192537

chr2 21192538 21192771

chr2 21192988 21194496

chr2 21194848 21196350

chr2 21196363 21197020

chr2 21197048 21197328

chr2 21197633 21199700

chr2 21199703 21201729

chr2 21203198 21204382

chr2 21204458 21207643

chr2 21207918 21209072

chr2 21209083 21209940

chr2 21209948 21214984

chr2 21214998 21218240

chr2 21218538 21220666

chr2 21220693 21220940

chr2 21221083 21221156

chr2 21221273 21222325

chr2 21222333 21222579

chr2 21222618 21223655

chr2 21224118 21226217

chr2 21226218 21231821

chr2 21231828 21237238

chr2 21237243 21238883

chr2 21239163 21239580

chr2 21239908 21240007

chr2 21240023 21240846

chr2 21240848 21241385

chr2 21241388 21244832

chr2 21245118 21247214

chr2 21247508 21249124

chr2 21249413 21250182

chr2 21250463 21252031

chr2 21252388 21252968

chr2 21253383 21253455

chr2 21253463 21253530

chr2 21253553 21253626

chr2 21253773 21253895

chr2 21254078 21254151

chr2 21254223 21254464

chr2 21254763 21261193

chr2 21261493 21262440

chr2 21262523 21262802

chr2 21263173 21264807

chr2 21264813 21266741

chr2 21266758 21268803

chr2 21269088 21270171

chr2 21270198 21272223

chr2 21272238 21272375

chr2 21273123 21274077

chr2 21274213 21274352

chr2 21274568 21274705

chr2 21274738 21274871

chr2 21274918 21275591

chr2 21275608 21277182

chr2 21277238 21277543

chr2 21277863 21278027

chr2 21278033 21278112

chr2 21278158 21278636

chr2 21278658 21280337

chr2 21280613 21282933

chr2 21282998 21284318

chr2 21284623 21284916

chr2 21285048 21285430

chr2 21285438 21287147

chr2 21287163 21288095

chr2 21288118 21288327

chr2 21288398 21288507

chr2 21288513 21289958

chr2 21289968 21290049

chr2 21290058 21290545

chr2 21290595 21291853

chr2 21291865 21292213

chr2 21292495 21294515

chr2 21294520 21294691

chr2 21294705 21295293

chr2 21295310 21295381

chr2 21295430 21295548

chr2 21295550 21295727

chr2 21295730 21295994

chr2 21296000 21296177

chr2 21296205 21296492

chr2 21296520 21296658

chr2 21296685 21296991

chr2 21296995 21297062

chr2 21297405 21297780

chr2 21297800 21298244

chr2 21298315 21298555

chr2 21298570 21299472

chr2 21299535 21299719

chr2 21299760 21299857

chr2 21299875 21299982

chr2 21299990 21301072

chr2 21301100 21301400

chr2 21301405 21301484

chr2 21301575 21301740

chr2 21303460 21304469

chr2 21304475 21304556

chr2 21304570 21304646

chr2 21304665 21305052

chr2 21305110 21305793

chr2 21305825 21307041

chr2 21307205 21308282

chr2 21308300 21308583

chr2 21308650 21309196

chr2 21309385 21309837

chr2 21310165 21310792

chr2 21310820 21310984

chr2 21310990 21312629

chr2 21312925 21313385

chr2 21313390 21313630

chr2 21313690 21315781

chr2 21315790 21316074

chr2 21316145 21316253

chr2 21316455 21316532

chr2 21316535 21316675

chr2 21316685 21317702

chr2 21318005 21318957

chr2 21318980 21319744

chr2 21319745 21319875

chr2 21319880 21322014

chr2 21322025 21322795

chr2 21322800 21322875

chr2 21322915 21322992

chr2 21323080 21323183

chr2 21323245 21323746

chr2 21324015 21324085

chr2 21324155 21324355

chr2 21324655 21324724

chr2 21325145 21325996

chr2 21326015 21328715

chr2 21328745 21328918

chr2 21329200 21329399

chr2 21329735 21329811

chr2 21329825 21330421

chr2 21330425 21330639

chr2 21331390 21331479

chr2 21331505 21333294

chr2 21333310 21334466

chr2 21334485 21335012

chr2 21335310 21335943

chr2 21335945 21336226

chr2 21336235 21336841

chr2 21336850 21338232

chr2 21338505 21339661

chr2 21339935 21340152

chr2 21340425 21340520

chr2 21340540 21340615

chr2 21340640 21342595

chr2 21342670 21342841

chr2 21343110 21343673

chr2 21343680 21343754

chr2 21343880 21344022

chr2 21344320 21344426

chr2 21344470 21345135

chr2 21345155 21345294

chr2 21345335 21345743

chr2 21345780 21345879

chr2 21345880 21347250

chr2 21347780 21347892

chr2 21348025 21348137

chr2 21348150 21348299

chr2 21348370 21348860

chr2 21348900 21349245

chr2 21349510 21350040

chr2 21350065 21351155

chr2 21351485 21351648

chr2 21351685 21351812

chr2 21351985 21352685

chr2 21352745 21353011

chr2 21353020 21354643

chr2 21354665 21355086

chr2 21355105 21355180

chr2 21355190 21356234

chr2 21356250 21356783

chr2 21356795 21357171

chr2 21357180 21357866

chr2 21358100 21359208

chr2 21359230 21364969

chr2 21365035 21366014

chr2 21366040 21366762

chr2 21366795 21367839

chr2 21367855 21369351

chr2 21369370 21370308

chr2 21370410 21370535

chr2 21370540 21371934

chr2 21372105 21372269

chr2 21372275 21372488

chr2 21372775 21372908

chr2 21372930 21373027

chr2 21373030 21373955

chr2 21376725 21376859

chr2 21376860 21378526

chr2 21378560 21378910

chr2 21378965 21379564

chr2 21379580 21379788

chr2 21379885 21380312

chr2 21380330 21380431

chr2 21380445 21381634

chr2 21381640 21382019

chr2 21382020 21383247

chr2 21383525 21383982

chr2 21384015 21384410

chr2 21384525 21384661

chr2 21384705 21384990

chr2 21385125 21385231

chr2 21385240 21385685

chr2 21386015 21387657

chr2 21387670 21389036

chr2 21389045 21389314

chr2 21389320 21389846

chr2 21389855 21390366

chr2 21390385 21390491

chr2 21390590 21390969

chr2 21390970 21391171

chr2 21391175 21391339

chr2 21391355 21391570

chr2 21391580 21391682

chr2 21391695 21392175

chr2 21392925 21392999

chr2 21393560 21393729

chr2 21393790 21393962

chr2 21393985 21394063

chr2 21394170 21394762

chr2 21394775 21395350

chr2 21395405 21395510

chr2 21395530 21395865

chr2 21395875 21397228

chr2 21397360 21400307

chr2 21400375 21400486

chr2 21400610 21400687

chr2 21400700 21401510

chr2 21401520 21401935

chr2 21401950 21403029

chr2 21403045 21403817

chr2 21403825 21405955

chr2 21406105 21407646

chr2 21407655 21407934

chr2 21408050 21408224

chr2 21408230 21408696

chr2 21408700 21409845

chr2 21410020 21410089

chr2 21410130 21410206

chr2 21410230 21410362

chr2 21413830 21414344

chr2 21414350 21414615

chr2 21415025 21415196

chr2 21415370 21416144

chr2 21416155 21417748

chr2 21417760 21419088

chr2 21419145 21419376

chr2 21419530 21419643

chr2 21419645 21420455

chr2 21420460 21421154

chr2 21421170 21421542

chr2 21421550 21423849

chr2 21423850 21424198

chr2 21424215 21425125

chr2 21425130 21425720

chr2 21425735 21426076

chr2 21426275 21426383

chr2 21426685 21427239

chr2 21427245 21427654

chr2 21427660 21428878

chr2 21429005 21429463

chr2 21429465 21430099

chr2 21430250 21430331

chr2 21430390 21431399

chr2 21437585 21437718

chr2 21438020 21438092

chr2 21438095 21439213

chr2 21439225 21439307

chr2 21439310 21439655

chr2 21439805 21439916

chr2 21439995 21440202

chr2 21440230 21440407

chr2 21440415 21441412

chr2 21441420 21442360

chr2 21442370 21444253

chr2 21444345 21444439

chr2 21444445 21445391

chr2 21445400 21448017

chr2 21448330 21449124

chr2 21449125 21449723

chr2 21449730 21450078

chr2 21450110 21450184

chr2 21450185 21450432

chr2 21450440 21450780

chr2 21450845 21451048

chr2 21451050 21452524

chr2 21452530 21455041

chr2 21455060 21455239

chr2 21455260 21457214

chr2 21457215 21457460

chr2 21457485 21458359

chr2 21458370 21458969

chr2 21458975 21460700

chr2 21460710 21462347

chr2 21462350 21462483

chr2 21463185 21463260

chr2 21464470 21464605

chr2 21464665 21467873

chr2 21467875 21469589

chr2 21469675 21469783

chr2 21469910 21470603

chr2 21470620 21471154

chr2 21471165 21471348

chr2 21471355 21471455

chr2 21471755 21471928

chr2 21471940 21474768

chr2 21474785 21475692

chr2 21475705 21477341

chr2 21477345 21480258

chr2 21480270 21482096

chr2 21482150 21483682

chr2 21483685 21484000

chr2 21484250 21484843

chr2 21484855 21485276

chr2 21485280 21485430

chr2 21485440 21489432

chr2 21489645 21494958

track name=target_region description="Target Regions"

chr2 20996301 21494945 chr2:20996301-21494945
