# Supplementary material for: Multiple rare and common variants in APOB gene locus associated with oxidatively modified low-density lipoprotein levels
Source: PLoS One. 2019 May 31;14(5):e0217620. doi: 10.1371/journal.pone.0217620 (PMC6544350; doi:10.1371/journal.pone.0217620)
Supplement: S1 Appendix — (PDF) [file pone.0217620.s008.pdf]

S1 Appendix

Khlebus E, et al. Multiple rare and common variants in APOB gene locus associated with oxidatively modified low-density lipoprotein levels.

Study design and subjects

Individuals for our work were selected from the study named "Approbation and implementation of new approaches to prevention, diagnosis, and treatment of atherosclerosis in outpatient settings by the example of the Western Administrative District of Moscow". The random recruitment of 776 patients from the out-patient hospitals of the Western Administrative District of Moscow (Russia) was performed from August to December 2009.

For selected 776 patients we measured the level of oxidatively modified low density lipoproteins (oxLDL). Then we performed genotyping and genome-wide association study (GWAS). After we selected for targeted sequencing those 48 individuals who had the lowest oxLDL levels (with the median of 35.29 U/dl) and 48 individuals with the highest ones (with the median of 118.35 U/dl). These groups differed significantly by oxLDL levels ($P = 3.15 \times 10^{-17}$ by χ^2 test).

In all subjects, anthropometric parameters (height and weight) were measured and body mass index was calculated. Smoking status (smoker or non-smoker) was assessed in all study participants.

Hypertension was defined for a mean systolic blood pressure of at least 140 mmHg and/or a mean diastolic pressure of at least 90 mmHg (repeated at least 2 times in different visits), or currently taking antihypertensive drugs. Blood pressure was measured after five minutes rest in the seated position with a mechanical tonometer after checking for the device accuracy. Diabetes mellitus was defined as a fasting serum glucose $\geq$ 7.0 mmol/l, glycosylated hemoglobin $\geq$ 6.5%, a history of diabetes mellitus or the use of blood glucose lowering agents.

Coronary artery disease was verified by medical records and physical examination, and, if necessary, a stress test was carried out. Myocardial infarction and stroke were verified by medical records.

The study was approved by the Ethics Committee of the National Medical Research Center of Cardiology. Written informed consent was obtained from the participants.

Laboratory tests

Venous blood sample was taken from subjects after they had fasted overnight. Total cholesterol (TC), triglycerides (TG), high-density lipoprotein cholesterol (HDL), apolipopritein A1, apolipoprotein B, high-sensitivity C-reactive protein (CRP), and lipoprotein (a) levels were measured using an automatic biochemistry analyzer ARCHITECT c8000 (Abbott Laboratories, USA). Low-density lipoprotein cholesterol (LDL) was calculated according to the Friedewald formula (1). LDL was estimated by direct method in the case of TG>4.5 mmol/l using the same analyser ARCHITECT c8000 (Abbott Laboratories, USA).

Oxidatively modified LDL levels measurement

The circulating serum oxLDL levels were defined in the blood serum containing 1 mg/ml EDTA as anticoagulant. Measurement was performed using the hard-phase immune-enzyme assay Oxidized LDL ELISA (Mercodia, Sweden) by spectrophotometric plate reader BioTek EL 808 (BioTek, USA). LDL was isolated from the blood of patients by differential ultracentrifugation in a NaBr gradient using Ti-50 rotor in a Beckman L-8 ultracentrifuge (Beckman, USA) according to the protocol (2).

Atherosclerosis progression is accompanied by oxidative stress, while malondialdehyde (MDA) and other low molecular weight dicarbonyls can accumulate in blood plasma, including MDA homologue glyoxal and MDA isomer methylglyoxal (3). Glyoxal and methylglyoxal, like MDA, can cause the atherogenic modification of LDL (3; 2; 4). Previously we have investigated the binding specificity of native and modified LDL to the monoclonal mAb-4E6 antibodies, used in the Oxidized LDL ELISA kits (Mercodia, Sweden), to determine what kind of modification we can measure (5). In short, LDL particles isolated from the blood of healthy donors were incubated for 3 hours with malonic dialdehyde (MDA), glyoxal and methylglyoxal and dialyzed for 12 hours at 4°C (4). It was revealed that MDA-modified LDL particles are preferentially bind with mAb-4E6

antibodies, whereas glyoxal-modified LDL and methylglyoxal-modified LDL practically do not interact with these antibodies (Fig. S1). Thus, in the present research we have studied not any oxidized LDL, but mainly MDA-modified LDL, due to the high specificity of the interaction of mAb-4E6 antibodies with MDA-modified LDL. Further under oxidatively modified LDL (oxLDL) we mean only these MDA-modified LDL (5).

Microarray-based genotyping

Blood samples of the patients were collected into tubes containing EDTA and stored at -20°C. Genomic DNA for all genetic procedures was extracted from blood using the Qiagen DNA blood mini kit (Qiagen, USA) following the manufacturer's guidelines. Genotyping was performed by using Cardio-MetaboChip (Illumina, USA), designed to test 196,725 SNPs identified through genome-wide meta-analyses for metabolic and atherosclerotic cardiovascular diseases and traits. The fluorescently stained chip was imaged by Illumina Bead Array Reader and the Bead Scan Software (Illumina, USA). GenomeStudio Genotyping Module (Illumina, USA) was used to analyze BeadArray data.

Quality control of genotyping results

Quality control was conducted on the raw genotyping data from 196,725 SNPs in DNA samples from 776 patients, using PLINK (v 1.07) (6). We performed it to filter out both unqualified samples and SNPs. The samples with low genotyping rate of $<90\%$ were excluded from further analysis (12 samples). SNPs were excluded according to the following criteria: (i) 250 SNPs were not mapped on autosomal chromosomes; (ii) 93,477 SNPs had a minor allele frequency <0.05; (iii) a total of 1,236 SNPs had a call rate <0.9 and (iv) for 58 markers genotype distributions clearly deviated from those expected by Hardy-Weinberg equilibrium ($P < 1.0 \times 10^{-5}$). Together, 101,704 SNPs in autosomal chromosomes passed the quality-control filters and were used for the GWAS. Next, an additional 32 unexpected duplicates or probable relatives were excluded based on pairwise identity by state according to their PI_HAT values in PLINK (all PI_HAT $>$ 0.185) (732 patients remained). Heterozygosity rates were calculated using PLINK, and more than 3 s.d. from the mean was used as the exclusion criterion (seven samples excluded). To determine whether our sequence variations were caused by the population stratification, we assayed genotyping data using the principal component analysis (PCA). The PCA revealed no evidence of differences in genetic ancestry between samples (Fig. S2). After all filtering steps, the data from remaining 725 individuals were used for the GWAS, total genotyping rate in remaining samples was 0.9976.

Target enrichment and DNA sequencing

For targeted sequencing 4 μg of genomic DNA was fragmented by ultrasound to the size of 150-250 bp using Covaris S220 (Covaris, USA) according to the manufacturer's protocol. The amount of DNA was measured with a fluorimeter Qubit 2.0 (Thermo Fisher Scientific, USA). The size distribution of DNA fragments was assessed with Agilent 2100 Bioanalyzer (Agilent Technologies, USA). Targeted sequencing was performed using the TargetSeq Custom Enrichment Kit (Thermo Fisher Scientific, USA) on the SOLiD 5500W system (Thermo Fisher Scientific, USA) according to the manufacturer's protocol. TargetSeq Custom Enrichment Kit was designed to target the region containing the complete genomic sequence of the *APOB* gene in locus 2p24-p23 (chr2: 20996301-21494945; GRCh37/hg19 reference human genome). The kit consists of 536 fragments accounting for a total of 391 833 bp. Unique probes were designed using the Sequence Search and Alignment by Hashing Algorithm (SSAHA) (7). Capture design coordinates are provided in Dataset S1.

Targeted sequencing data processing

At the end of the targeted sequencing on SOLiD 5500W system (Thermo Fisher Scientific, USA), all information was written into .xsq files. Raw reads from .xsq file were aligned to the GRCh37/hg19 reference human genome using LifeScope Genomic Analysis Software (Life Technologies, USA) for each sample individually, .bam files were generated. Then we used Samtools (8) for duplicate reads removal. SNPs calling was performed by LifeScope Genomic Analysis Software (Life Technologies, USA). We used ANNOVAR software (9) for annotation the detected variants. Functional effects of these variants were assessed with the SIFT (10) and PolyPhen-2 (11) algorithms.

To get coverage data we used powerful toolset for genome arithmetic BedTools (12), which allows to determine the read coverage for each position. We filtered variants by coverage using 10x coverage threshold. Base was considered not covered if it was supported by less than 10 reads (less than 5 for alternative allele). Each base with low-quality (Phred score < 30) sequence was also removed. In our work we used Phred score value as a cutoff. The Phred mapping quality score represents the probability that the read was mapped incorrectly, value 30 means that read assigned a Phred mapping quality with this score has a 1 in 1000 chance of being misaligned (13). Further data were processed with custom Perl scripts and R statistical software v. 3.0.1 (http://www.rproject.org). As a result, for each patient files with SNPs, its coordinates, coverage and other characteristics were generated. Variations detected in *APOB* were named using cDNA sequence RefSeq accession number NM_000384.2. Amino acid sequence changes in ApoB are described according to the National Center for Biotechnology Information reference sequence (ApoB, RefSeq accession no. NP_000375.2).

Multiple protein alignment

Multiple protein alignment for evolutionary conservation analysis was obtained using MUSCLE with default settings (14) and visualized using Jalview (15). The following NCBI sequences were used for analysis: *Homo sapiens* (human) - P04114.2, *Pan troglodytes* (chimpanzee) - XP_515323.3, *Macaca mulatta* (Rhesus monkey) - XP_001097500.2, *Oryctolagus cuniculus* (rabbit)- XP_008253005.1, *Bos taurus* (cow) - DAA24500.1, *Canis lupus familiaris* (dog)- XP_005630653.1, *Echinops telfairi* (hedgehog) - XP_004696455.1, *Mus musculus* (house mouse) - E9Q414.1, *Rattus norvegicus* (rat) - Q7TMA5.1, *Gallus gallus* (chicken) - ABF70173.1, *Xenopus tropicalis* (frog) - XP_002934538.2, *Danio rerio* (zebrafish) - XP_694827.7.

Variant association testing

The data from targeted sequencing were analyzed by statistical methods developed for variants association testing: weighted-sum test (WST) (16), sequence kernel association test (SKAT) (17), SKAT-optimized (SKAT-O) (18), SKAT for combined common and rare variants analysis (SKAT-C) (19). For the analysis of rare and low-frequency variants by WST we used the custom script in programming language R, for methods SKAT, SKAT-O and SKAT-C R-package (20). To select a subgroup of associated variants we used BE-SKAT (21). We used ADA method (22) at the SNP-level to test for two-sided critical region, which was downloaded from the authors' website http://homepage.ntu.edu.tw/ linwy/ADAprioritized.html. Methods WST and ADA were conducted with 1000 permutations. In SKAT-based tests, the standard weights (17) were used and the sample size adjustment was applied. Age, sex, smoking status, body mass index, waist, HDL, CRP, lipoprotein (a), hypertension, myocardial infarction, diabetes mellitus, stroke and statins were used as covariates. To select a subgroup of associated variants, as suggested at (23), we used the elastic net (24) from R-package (25) with AUC-ROC-based cross-validation. For that purpose patients were randomly divided into 9 subgroups.

Small-sample (small group of patients) adjustment and presence of covariates make it impossible to use the BE-SKAT implementation proposed by its authors. Small-sample adjustment is evaluated by permutation method where permutation is defined by random seed. Different seeds can cause different results of BE-SKAT. To take this into account permutation we performed BE-SKAT a hundred times with different random seeds. Then we selected variants that were included in at least 95% of BE-SKATs' results. The other problem of small sample size is lack of robustness to small random noise. To reduce them we recommend to stop backward elimination procedure when reduction of P-value is small enough. In our implementation we stop the algorithm if P-value on current step is less than P-value on previous step by 5% or less. For example we chose group of variants with P-value close to the least, but not the least.

Minor allele frequency (MAF) for each variant was received from the Exome Aggregation Consortium (ExAC) database (http://exac.broadinstitute.org/). Variants with MAF$<$0.01 were defined as rare, variants with MAF in 0.01–0.05 were defined as low-frequency, the rest were considered as common. For novel variants without MAF data in the existing databases we defined MAF equal to the minimal MAF found for our variants set.

We considered an additive genetic model with an encoding: 0-wild type, 1 – heterozygote, 2 – minor homozygote. The high and low levels of oxLDL were denote as 1 an 0 respectively. We define the significance level of 0.05.

Data visualization

The Manhattan and quantile-quantile plots were generated by 'qqman' package in R (https://www.r-project.org/). Regional association plot was drawn using genome build GRCh37/hg19 and local linkage disequilibrium information from 1000 Genomes Project (reference version: Nov 24, 2014; EUR) by LocusZoom web-based plotting tool (26). LocusZoom can be found at http://csg.sph.umich.edu/locuszoom. The heatmap matrix of pairwise linkage disequilibrium statistics for variants with nonmissing SNP ID data was created by LDlink web-based application (available at http://analysistools.nci.nih.gov/LDlink/). Haplotype data from Phase 3 (Version 5, CEU) of the 1000 Genomes Project and genome build GRCh37/hg19 were used.

References

[1] W. T. Friedewald, R. I. Levy, and D. S. Fredrickson, "Estimation of the concentration of low-density lipoprotein cholesterol in plasma, without use of the preparative ultracentrifuge," *Clinical chemistry*, vol. 18, no. 6, pp. 499–502, 1972.

[2] V. Lankin, G. Konovalova, A. Tikhaze, K. Shumaev, E. Kumskova, and M. Viigimaa, "The initiation of free radical peroxidation of low-density lipoproteins by glucose and its metabolite methylglyoxal: a common molecular mechanism of vascular wall injure in atherosclerosis and diabetes," *Molecular and cellular biochemistry*, vol. 395, no. 1-2, pp. 241–252, 2014.

[3] V. Z. Lankin and A. K. Tikhaze, "Role of oxidative stress in the genesis of atherosclerosis and diabetes mellitus: a personal look back on 50 years of research," *Current aging science*, vol. 10, no. 1, pp. 18–25, 2017.

[4] V. Lankin, A. Tikhaze, V. Kapel'ko, G. Shepel'kova, K. Shumaev, O. Panasenko, G. Konovalova, and Y. N. Belenkov, "Mechanisms of oxidative modification of low density lipoproteins under conditions of oxidative and carbonyl stress," *Biochemistry (Moscow)*, vol. 72, no. 10, pp. 1081–1090, 2007.

[5] E. Y. Khlebus, N. Meshkov, V. Lankin, A. Orlovsky, V. Kiseleva, N. Shcherbakova, . Zharikova, I. Ershova, . Tikhaze, B. Yarovaya, I. Chazova, and S. Boytsov, "Lipid profile and genetic markers associated with the level of oxidized low density lipoproteides," *Russian Journal of Cardiology*, vol. 10, no. 150, pp. 49–54, 2017.

[6] S. Purcell, B. Neale, K. Todd-Brown, L. Thomas, M. A. Ferreira, D. Bender, J. Maller, P. Sklar, P. I. De Bakker, M. J. Daly, *et al.*, "Plink: a tool set for whole-genome association and population-based linkage analyses," *The American Journal of Human Genetics*, vol. 81, no. 3, pp. 559–575, 2007.

[7] Z. Ning, A. J. Cox, and J. C. Mullikin, "Ssaha: a fast search method for large dna databases," *Genome research*, vol. 11, no. 10, pp. 1725–1729, 2001.

[8] H. Li, B. Handsaker, A. Wysoker, T. Fennell, J. Ruan, N. Homer, G. Marth, G. Abecasis, and R. Durbin, "The sequence alignment/map format and samtools," *Bioinformatics*, vol. 25, no. 16, pp. 2078–2079, 2009.

[9] K. Wang, M. Li, and H. Hakonarson, "Annovar: functional annotation of genetic variants from high-throughput sequencing data," *Nucleic acids research*, vol. 38, no. 16, pp. e164–e164, 2010.

[10] P. Kumar, S. Henikoff, and P. C. Ng, "Predicting the effects of coding non-synonymous variants on protein function using the sift algorithm," *Nature protocols*, vol. 4, no. 7, p. 1073, 2009.

[11] I. A. Adzhubei, S. Schmidt, L. Peshkin, V. E. Ramensky, A. Gerasimova, P. Bork, A. S. Kondrashov, and S. R. Sunyaev, "A method and server for predicting damaging missense mutations," *Nature methods*, vol. 7, no. 4, p. 248, 2010.

[12] A. R. Quinlan and I. M. Hall, "Bedtools: a flexible suite of utilities for comparing genomic features," *Bioinformatics*, vol. 26, no. 6, pp. 841–842, 2010.

[13] B. Ewing, L. Hillier, M. C. Wendl, and P. Green, "Base-calling of automated sequencer traces usingphred. i. accuracy assessment," *Genome research*, vol. 8, no. 3, pp. 175–185, 1998.

[14] R. C. Edgar, "Muscle: multiple sequence alignment with high accuracy and high throughput," *Nucleic acids research*, vol. 32, no. 5, pp. 1792–1797, 2004.

[15] A. M. Waterhouse, J. B. Procter, D. M. Martin, M. Clamp, and G. J. Barton, "Jalview version 2—a multiple sequence alignment editor and analysis workbench," *Bioinformatics*, vol. 25, no. 9, pp. 1189–1191, 2009.

[16] B. E. Madsen and S. R. Browning, "A groupwise association test for rare mutations using a weighted sum statistic," *PLoS genetics*, vol. 5, no. 2, p. e1000384, 2009.

[17] M. C. Wu, S. Lee, T. Cai, Y. Li, M. Boehnke, and X. Lin, "Rare-variant association testing for sequencing data with the sequence kernel association test," *The American Journal of Human Genetics*, vol. 89, no. 1, pp. 82–93, 2011.

[18] S. Lee, M. J. Emond, M. J. Bamshad, K. C. Barnes, M. J. Rieder, D. A. Nickerson, D. C. Christiani, M. M. Wurfel, X. Lin, N. G. E. S. Project, *et al.*, "Optimal unified approach for rare-variant association testing with application to small-sample case-control whole-exome sequencing studies," *The American Journal of Human Genetics*, vol. 91, no. 2, pp. 224–237, 2012.

[19] I. Ionita-Laza, S. Lee, V. Makarov, J. D. Buxbaum, and X. Lin, "Sequence kernel association tests for the combined effect of rare and common variants," *The American Journal of Human Genetics*, vol. 92, no. 6, pp. 841–853, 2013.

[20] S. Lee, L. Miropolsky, and M. Wu, "Skat: Snp-set (sequence) kernel association test r package version 1.1. 2," 2015.

[21] I. Ionita-Laza, M. Capanu, S. De Rubeis, K. McCallum, and J. D. Buxbaum, "Identification of rare causal variants in sequence-based studies: methods and applications to vps13b, a gene involved in cohen syndrome and autism," *PLoS genetics*, vol. 10, no. 12, p. e1004729, 2014.

[22] K. Yu, Q. Li, A. W. Bergen, R. M. Pfeiffer, P. S. Rosenberg, N. Caporaso, P. Kraft, and N. Chatterjee, "Pathway analysis by adaptive combination of p-values," *Genetic epidemiology*, vol. 33, no. 8, pp. 700–709, 2009.

[23] A. Malovini, R. Bellazzi, C. Napolitano, and G. Guffanti, "Multivariate methods for genetic variants selection and risk prediction in cardiovascular diseases," *Frontiers in cardiovascular medicine*, vol. 3, p. 17, 2016.

[24] H. Zou and T. Hastie, "Regularization and variable selection via the elastic net," *Journal of the Royal Statistical Society: Series B (Statistical Methodology)*, vol. 67, no. 2, pp. 301–320, 2005.

[25] J. Friedman, T. Hastie, and R. Tibshirani, "glmnet: Lasso and elastic-net regularized generalized linear models," *R package version*, vol. 1, no. 4, 2009.

[26] R. J. Pruim, R. P. Welch, S. Sanna, T. M. Teslovich, P. S. Chines, T. P. Gliedt, M. Boehnke, G. R. Abecasis, and C. J. Willer, "Locuszoom: regional visualization of genome-wide association scan results," *Bioinformatics*, vol. 26, no. 18, pp. 2336–2337, 2010.
