# Supplementary material for: Multiple rare and common variants in APOB gene locus associated with oxidatively modified low-density lipoprotein levels
Source: PLoS One. 2019 May 31;14(5):e0217620. doi: 10.1371/journal.pone.0217620 (PMC6544350; doi:10.1371/journal.pone.0217620)
Supplement: S1 Table — (PDF) [file pone.0217620.s009.pdf]

Parameter	r (Spearman)	P-value
Total cholesterol	0.54	9.62×10^{-56}
Triglycerides	0.33	3.50×10^{-20}
Low-density lipoprotein cholesterol	0.55	4.49×10^{-59}
High-density lipoprotein cholesterol	-0.08	0.025
High-sensitivity C-reactive protein	0.14	2.19×10^{-4}
Lipoprotein (a)	0.02	0.533
Apolipoprotein A1	0.01	0.709
Apolipoprotein B	0.61	2.34×10^{-68}
Total stenosis	0,11	5.54×10^{-3}
Plaque number	0,11	6.79×10^{-3}
Mean intima-medial thickness	0,11	7.02×10^{-3}

Table S1: Correlation of oxLDL levels with biochemical parameters and ultrasound markers of atherosclerosis.
