# Supplementary material for: Multiple rare and common variants in APOB gene locus associated with oxidatively modified low-density lipoprotein levels
Source: PLoS One. 2019 May 31;14(5):e0217620. doi: 10.1371/journal.pone.0217620 (PMC6544350; doi:10.1371/journal.pone.0217620)
Supplement: S2 Table — All SNPs are localized on Chromosome 2 in APOB gene region. (PDF) [file pone.0217620.s010.pdf]

Genomic position (GRCh37/hg19)	SNP ID	MAF (1000Genomes)	Annotation	SIFT/ PolyPhen-2	Amino acid change	P-value
21198900	rs11902417	0.385383	intergenic			3.60×10^{-9}
21203877	rs10184054	0.371006	intergenic			7.08×10^{-9}
21206183	rs6754295	0.40595	intergenic			7.85×10^{-9}
21204025	rs6544366	0.371605	intergenic			8.16×10^{-9}
21205502	rs4564803	0.386182	intergenic			8.55×10^{-9}
21208211	rs7557067	0.370008	intergenic			9.54×10^{-9}
21225281	rs1042034	0.629593	missense	T / B	p.Ser4338Asn	1.13×10^{-8}
21226560	rs2678379	0.610423	intronic			1.14×10^{-8}
21231524	rs676210	0.366214	missense	D / D	p.Pro2739Leu	1.14×10^{-8}
21237544	rs673548	0.385783	intronic			1.14×10^{-8}
21193946	rs6728178	0.384984	intergenic			1.23×10^{-8}
21142994	rs4296389	0.534145	intergenic			7.29×10^{-8}
21240031	rs11680233	0.611821	intronic			8.61×10^{-8}
21240364	rs11126598	0.613419	intronic			2.18×10^{-7}

Table S2: Fourteen the most significant SNPs obtained by GWAS for oxLDL levels in 725 samples. All SNPs are localized on Chromosome 2 in *APOB* gene region.

SIFT score: T - Tolerated, D - Deleterious; PolyPhen-2 score: B - Benign, D - Probably damaging.
